# Tackling Challenges of Textile Recycling: Color Removal from Cotton Textile Waste via Sequential Reductive–Oxidative Bleaching

**DOI:** 10.1021/acsomega.6c02988

**Published:** 2026-08-13

**Authors:** Shubhajit Dutta, Md. Reazuddin Repon, Inge Schlapp-Hackl, Tapani Vuorinen, Ali Tehrani-Bagha

**Affiliations:** Department of Bioproducts and Biosystems, School of Chemical Engineering, 174277 Aalto University, Vuorimiehentie 1, Espoo 02150, Finland

## Abstract

An effective pathway for textile recycling could be the successful integration of the textile waste into a closed-loop system; however, one of the key challenges is the removal of color from textile waste. In this study, we developed a two-step reductive-oxidative treatment to convert reactive-dyed textile waste into a white feedstock suitable for textile recycling. The process includes a reductive stage using Na_2_S_2_O_4_, followed by oxidative bleaching with H_2_O_2_. Colorimetric, mechanical, morphological, polymeric, elemental, and thermal analyses were performed using standard methods to evaluate the characteristics of the color-stripped fabrics. Under optimized conditions, up to 99.3% color stripping was achieved, along with high lightness (L* = 89.1) and whiteness index (WI = 87.6). The average tensile strength retention of 87.2%, maximum weight loss of 3.5%, limited surface fibrillation observed under scanning electron microscopy, and a minimum degree of polymerization of 1404 suggested that only limited cellulose degradation occurred, allowing the treated materials to retain the properties required for subsequent chemical recycling. Bulk and surface elemental analyses further confirmed a significant reduction in nitrogen content and the complete removal of sulfur, demonstrating effective dye removal and the restoration of the fabric to a composition closely resembling that of the undyed material, while the data from X-ray diffraction revealed only minor changes in the crystallinity index, indicating that the polymer structure remained stable. Thermogravimetric analysis likewise indicated that the thermal profile of the fabrics after dye removal was restored to that of the undyed fabric. The excellent redyeability and uniform color uptake of the color-stripped fabrics indicated their promising potential to be reintroduced into circular textile systems through either chemical recycling into new textile products or mechanical recycling via shredding and blending with virgin fibers.

## Graphical abstract

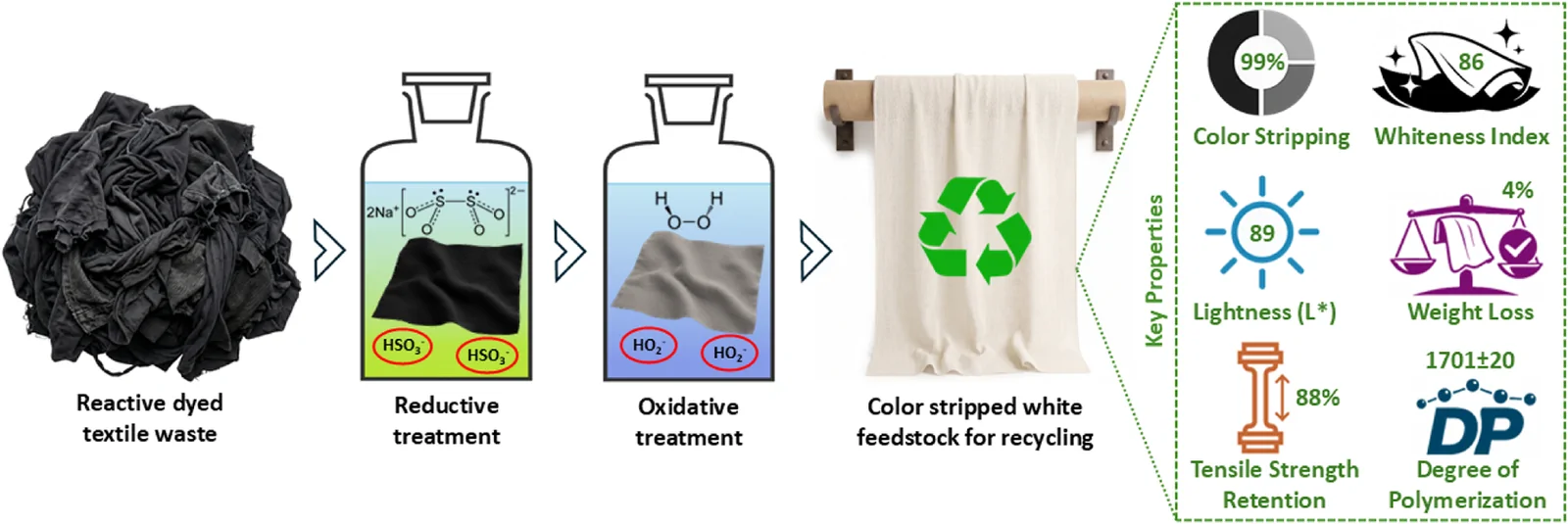

## 1 Introduction

In a world striving for sustainability, the textile industry remains deeply rooted in a linear make–wear–discard model, contributing significantly to environmental degradation through resource-intensive production and escalating postconsumer waste. In the European Union, textile consumption increased from 17 to 19 kg per person between 2019 and 2022, contributing to the generation of 6.95 million tonnes of postconsumer textile waste in 2022.^1^ On the other hand, global textile waste reached 120 million metric tons in 2024. Nearly 80% of discarded clothes ended up in landfills or incinerators, while 1% was neither reusable nor recyclable, only 12% was reused, and 7% was technically recyclable. As a result, the amount of global textile waste is projected to exceed 150 million metric tons annually within the next 5 years, intensifying the sector’s ecological footprint.^2^ Addressing this challenge requires a shift toward circular economy principles, emphasizing the reuse, recycling, and upcycling of materials to extend product lifecycles and reduce the demand for virgin resources. In a circular textile system, waste is transformed into raw materials for new products, minimizing environmental issues while supporting economic growth and ecological balance. However, recycling dyed textile waste remains a critical hurdle, as persistent dyes and finishes hinder efficient fiber recovery, yarn regeneration, and the production of high-quality recycled products.

Among the various challenges, the most critical is that shredding cotton textile waste leads to unpredictable mélange shades, making it difficult to obtain a white and uniform feedstock for recycling. Additionally, reactive dyes pose a major barrier to textile-to-textile recycling due to their widespread use in cotton fabrics and their strong covalent bonding with cellulose, which complicates post-use processing. Besides this, extensive research has also been conducted on dye removal from aqueous systems, including adsorption and catalytic degradation approaches.^3,4^ However, comparatively less attention has been given to color removal directly from dyed textile substrates, which is critical for textile-to-textile recycling. Consequently, color stripping, the deliberate removal of dyes from fibers, has emerged as a critical step in recycling reactive-dyed textiles. Recent research has explored various chemical color-stripping approaches for cotton fabrics dyed with reactive dyes, including oxidative,^5–12^ reductive,^13–17^ sequential treatments,^18–21^ biological,^22,23^ microwave-assisted,^24–27^ and solvent-based^28^ methods. A comparative evaluation of the performance of these methods on reactive-dyed cotton textile waste, together with the results obtained in the present study, is provided in Table S1. Although several of these approaches have demonstrated promising decolorization efficiencies, many still require high chemical dosages, elevated temperatures, and prolonged treatment times, often resulting in significant loss of fiber strength. To address these process parameters, bleaching agents commonly used in the pulp and paper industry could offer a promising solution. Pulp bleaching agents, such as sodium dithionite (Na_2_S_2_O_4_), hydrogen peroxide (H_2_O_2_), sodium hydroxide (NaOH), sodium chlorite (NaClO_2_), chlorine dioxide (ClO_2_), peracetic acid (CH_3_CO_3_H), and ozone (O_3_), can potentially be used for textile bleaching and wastewater treatments.^29–32^

Song et al. employed NaOH to remove reactive dyes from cotton fabrics.^13^ The process was conducted using 80 g/L NaOH, maintaining M:L of 1:100 at 90 °C for 90 min, which resulted in 86% color stripping. In the same study, a sequential treatment (NaOH-Na_2_S_2_O_4_) was introduced, and 98.5% color stripping was achieved. However, this approach involves high chemical consumption and extensive use of fresh water and has only been demonstrated for single-reactive dyed cotton fabric. In another study, Na_2_S_2_O_4_–H_2_O_2_ sequential color stripping of vinyl sulfone-based reactive-dyed cotton fabric using 30 g/L Na_2_S_2_O_4_ at 90 °C, followed by 40 g/L hydrogen peroxide at 70 °C, achieved 97.8% dye removal; however, the fiber damage was considerably high.^18^ Bigambo et al. implemented two different three-step processes, acid/dithionite/peroxide and acid/alkali/peroxide treatments, to remove reactive dyes from cotton, achieving 82% and 99% color stripping, respectively, for anthraquinone-based Reactive Blue 19.^33^ In another study, a three-step sequence (HCl–H_2_O_2_–Na_2_S_2_O_4_) was introduced, resulting in 88% color stripping, especially for Reactive Turquoise CLB.^27^ However, both processes require massive amounts of chemicals, are time-consuming, and result in a significant loss of tensile strength. He et al.^16^ introduced a UV–Na_2_S_2_O_4_ system to remove commercial reactive dyes from cotton. They investigated 15 different reactive dyes and reported a wide range of performances, with color stripping efficiencies varying between 43% and 92%. These results indicate that Na_2_S_2_O_4_ alone is not sufficiently effective for removing reactive dyes, even when combined with UV irradiation. Furthermore, this method has only been demonstrated on samples dyed with a single reactive dye, not a mixture of reactive dyes.^16^ However, to the best of our knowledge, no studies have reported the removal of mixed reactive dyes from cotton textile waste using sequential reductive–oxidative treatment.

Despite the growing research on dye removal from cellulosic textiles, existing approaches remain limited by high chemical demand, process complexity, and insufficient preservation of fiber integrity. Moreover, most studies focus on single reactive dyes under laboratory conditions, leaving a significant gap in addressing mixed reactive-dyed textile waste that is representative of real postconsumer materials. To bridge this gap, the present study introduces a two-step reduction–oxidation bleaching process designed to effectively strip color from reactive-dyed textile waste while maintaining the cellulose structure and mechanical strength. The process performance was evaluated through colorimetric, mechanical, chemical, morphological, and thermal analyses to comprehensively assess the treatment’s efficiency and material compatibility. This study introduces a viable approach to convert colored textile waste into a white, redyeable feedstock for fiber-to-fiber recycling, supporting circular textile manufacturing.

## 2 Experimental Information

### 2.1 Materials

An undyed white 100% cotton woven fabric (considered as the baseline/standard for this study) and four other fabrics dyed with different reactive dyes (details of the dyes are provided in Table 1) used in this study were provided by Elyaf Tekstil, Türkiye. The density of the yarns in the fabrics was 62 warp/cm and 55 weft/cm, and all fabrics had a uniform areal density of 94 g/m^2^.

**Table 1:** Color Index (C.I.) Number, Reactive Groups, Dye Concentration (%) of Each Reactive Dye, and Total Dye Weight (%) of Dyed Fabrics (DG, GR, BL, NV)

Dyed fabric	C.I. number	Reactive groups	Dye concentration (%)	Total dye weight (%)
**Dark Green (DG)**	Reactive Red 195	Monochlorotriazine & Vinyl Sulfone	0.2	3.6
	Reactive Blue 222		2.0	
	Reactive Yellow 160		1.4	
**Gray (GR)**	Reactive Yellow 145	Monochlorotriazine & Vinyl Sulfone	0.6	2.3
	Reactive Blue 221		1.1	
	Reactive Red 195		0.6	
**Black (BL)**	Reactive Black 5	Vinyl Sulfone	2.5	7.5
	Reactive Orange 16			
	Reactive Orange 107			
	Reactive Black Mix	Monochlorotriazine	5.0	
**Navy (NV)**	Reactive Extra Navy D	Monochlorotriazine & Vinyl Sulfone	2.2	4.5
	Reactive Red 195		1.0	
	Reactive Yellow 145		1.3	

Reactive dyes with concentration (%), chemical structures, molecular formulas, molecular weights, and CAS numbers are mentioned in Table S2. Sodium dithionite (Na_2_S_2_O_4_, 85%, CAS: 7775–14–6), hydrogen peroxide solution (H_2_O_2_, 50%; stabilized, CAS: 7722–84–1), sodium hydroxide (NaOH, 98%, CAS: 1310–73–2), glacial acetic acid (CH_3_COOH, 100%, CAS: 64–19–7), sodium carbonate (Na_2_CO_3_, ≥99.5%, CAS: 497–19–8), Glauber’s salt (Na_2_SO_4_·10H_2_O, ≥99.0%, CAS: 7727–73–3) were sourced from Sigma–Aldrich, Finland. Three commercially available reactive dyes (Bezactive Red S-3B 150, Bezactive Yellow S-3R 150, and Bezactive Blue S-GLD 150) were obtained from CHT Switzerland AG. A standard soaping agent (ISO 6330:2021, ECE reference detergent 3) without an optical brightening agent or enzyme was provided by SDC Enterprise, UK. All chemicals were of analytical grade and used as received without purification. Millipore water was used throughout the experimental work. All experiments and analyses were conducted in triplicate (*n* = 3) to ensure reproducibility and reliability of the results.

### 2.2 Methods

#### 2.2.1 Aging of Dyed Fabrics

All dyed fabrics were subjected to an aging process following 10 consecutive washing-drying cycles in a washing machine and a dryer. A standard soaping agent was used under controlled conditions for 1 h at 40 °C during each washing cycle, and a dryer was used for 30 min at 70 °C during each drying cycle.^34^ This aging process replicates the cumulative effects of repeated laundering over a fabric’s lifespan, mimicking postconsumer textile waste.

#### 2.2.2 Optimization Method

To systematically evaluate the influence of process variables, a one-factor-at-a-time approach was employed as an exploratory method for parameter tuning. In this approach, individual parameters were varied sequentially while keeping all other conditions constant, allowing the assessment of their independent effects on color removal performance. For the reductive treatment using Na_2_S_2_O_4_, the investigated parameter ranges included concentration (10–40 g/L), pH (9–12), temperature (40–100 °C), treatment time (30–120 min), and material-to-liquor (M:L) ratio (1:10–1:40). Similarly, for the oxidative treatment using H_2_O_2_, the parameters studied were concentration (1–7 g/L), pH (9–12), temperature (30–120 °C), treatment time (30–120 min), and M:L ratio (1:10–1:40). The selection of these ranges was based on preliminary trials and relevant literature.

#### 2.2.3 Color Stripping Process

A sequential reductive-oxidative treatment was performed on the dyed fabrics. In the first step, the reduction treatment of the dyed fabrics was conducted using 20 g/L Na_2_S_2_O_4_ at 60 °C for 60 min, maintaining an M:L ratio of 1:20 and a pH of 12. The pH of the reaction bath was controlled using 2 g/L NaOH. An IR sample dyeing machine (Testex TD 130, China) was used for this process. Soaping was followed by a 2 g/L standard soaping agent at 50 °C for 10 min to remove unfixed dye from the fiber surface. A neutralization process was then conducted using 2 mL/L glacial acetic acid to neutralize the fabrics. In the second step, oxidative treatment was performed using 1 g/L H_2_O_2_ at 90 °C for 60 min, maintaining the same M:L ratio. The pH of the bath was 11 and was controlled with 0.5 g/L NaOH. A Lauda Hydro Shaking Water Bath (Germany) was used for the oxidation process. Soaping and neutralization processes were conducted accordingly. A stepwise process diagram of the reductive–oxidative color stripping is presented in Figure S1. Finally, the color-stripped fabrics were oven-dried at 60 °C for 20 min and kept in the conditioning room for 24 h. Table 2 presents all the fabrics, including undyed, dyed, and stripped, with distinct identification for each.

**Table 2:** Identification of Undyed, Dyed, and Color-Stripped Fabrics

Sample	Condition	Identification
**White (WH)**	Undyed (Ud)	WH_Ud_
**Dark Green (DG)**	Dyed (Dy)	DG_Dy_
	Reductive stripped (Rd)	DG_Rd_
	Reductive-oxidative stripped (Rd-Ox)	DG_Rd‑Ox_
**Gray (GR)**	Dyed (Dy)	GR_Dy_
	Reductive stripped (Rd)	GR_Rd_
	Reductive-oxidative stripped (Rd-Ox)	GR_Rd‑Ox_
**Black (BL)**	Dyed (Dy)	BL_Dy_
	Reductive stripped (Rd)	BL_Rd_
	Reductive-oxidative stripped (Rd-Ox)	BL_Rd‑Ox_
**Navy (NV)**	Dyed (Dy)	NV_Dy_
	Reductive stripped (Rd)	NV_Rd_
	Reductive-oxidative stripped (Rd-Ox)	NV_Rd‑Ox_

#### 2.2.4 Redyeing Process

Redyeing was performed in the IR sample dyeing machine using a mixed reactive dye (1% o.w.f.) composed of Bezactive Red S-3B 150 (0.67%), Yellow S-3R 150 (0.30%), and Blue S-GLD 150 (0.03%). The dye bath (M:L = 1:20) contained 40 g/L Glauber’s salt and 15 g/L Na_2_CO_3_, and dyeing was conducted at 60 °C for 60 min under constant agitation. Four color-stripped fabrics, alongside undyed reference cotton (WH_Ud_) were dyed. After dyeing, the fabrics were rinsed, soaped at 80 °C for 10 min in 2 g/L ECE reference detergent, washed with warm water, and air-dried.

#### 2.2.5 Characterization

The changes in color strength (K/S), lightness (L*), and whiteness index (WI) of the fabrics before and after stripping were considered to assess the colorimetric properties of the fabrics. The X-Rite Ci7600 spectrophotometer, with a D65 light source, wavelength 360–780 nm, and a 10° observer, was used, and the maximum wavelength (λ_max_) was considered to measure the K/S. The measurements of the reflectance of the fabrics were repeated five times from five different spots on each of the fabric surfaces; and each time during measurement, the fabrics were folded in four layers to maintain uniform opacity. Following eq 1, the color-stripping percentage of the fabrics was calculated using the K/S values of the dyed and stripped fabrics. To present the color difference between the undyed white and the stripped fabrics, ΔE_CMC_ was extracted from the spectrophotometer.^35^ In addition to calculating the color-stripping percentage, the L* and WI of the fabrics were also considered. The L* of all the fabrics was collected from the spectrophotometer analysis, and the WI was calculated using eq 2, according to the Chinese national standard FZ/T51001–2009.^13^ The weight loss% was calculated using eq 3.

1 $$\mathrm{Color stripping}\left(\%\right)=\left(1-\frac{{\left(\frac{K}{S}\right)}_{\mathrm{stripped}}}{{\left(\frac{K}{S}\right)}_{\mathrm{dyed}}}\right)\times 100$$

2 $$\mathrm{Whiteness index}=100-\sqrt{{\left(100-L^{*}\right)}^{2}+{\left(a^{*}\right)}^{2}+{\left(b^{*}\right)}^{2}}$$

3 $$\mathrm{Weight Loss}\left(\%\right)=\left(\frac{{\mathrm{Weight}}_{\mathrm{dyed}}-{\mathrm{Weight}}_{\mathrm{stripped}}}{{\mathrm{Weight}}_{\mathrm{dyed}}}\right)\times 100$$

ISO 13934–1 strip method was followed to measure the tensile strength of the fabrics using a universal tester (Instron 4204). As all the fabrics were woven, the tensile strength of each fabric was measured in both the warp and weft directions. Subsequently, the tensile strength retention%^16^ was calculated using eq 4.

$$\mathrm{Tensile strength retention}\left(\%\right)=\left(\frac{{\mathrm{Tensile Strength}}_{s\mathrm{tripped}}}{{\mathrm{Tensile Strength}}_{\mathrm{dyed}}}\right)\times 100$$ 4

The crystallinity was measured using X-ray diffraction with a Xenocs Xeuss 3.0 diffractometer, which features a 2D Dectris Eiger2 R 1 M detector (sample-to-detector distance: 56 mm). CuKα radiation with a wavelength of 1.54189 Å was employed, operating in transmission mode at 50 kV and 0.6 mA. The scattering profiles were corrected based on blank runs. The crystallinity index (CI) was computed as the ratio of the total intensity area to the estimated background intensity using eq 5.^36^

5 $$\mathrm{Crystallinity Index}\left(\%\right)=100\cdot \frac{\int I\left(\theta \right)d\theta -\int I_{\mathrm{bkg}}\left(\theta \right)d\theta }{\int I\left(\theta \right)d\theta }$$

Where I­(θ) is the total intensity determined by a Savitzky–Golay filter with a window size of 29 and a polynomial order of 1, and I_bkg_(θ) is the background profile estimated with a window size of 201 and a polynomial order of 1.

Morphological changes were observed using a Zeiss Sigma VP SEM (scanning electron microscope) with an accelerating voltage of 4 kV. To enhance conductivity, the surface of all the fabrics was coated with gold–palladium (80/20) for 90 s using a Q150R Sputter Coater (Quorum Technologies).^37^

The intrinsic viscosity (η) of the ground (milled with Wiley Mini Mill 475-A) fabrics was determined by dissolving them in a cupri-ethylenediamine (CED) solution, according to the ISO 5351-1 standard. Subsequently, to calculate the degree of polymerization (DP), eq 6 is followed using the intrinsic viscosity (η) of the fabrics.

6 $$\mathrm{Degree of Polymerization}=e^{\frac{\ln\left[\eta \right]+\ln\left(0.439\right)}{0.76}}$$

Elemental analysis of the fabrics was performed using a Thermo Flash Smart CHNSO elemental analyzer (Thermo Scientific). For each analysis, all of the fabrics had three replicates. During the CHNS analysis, using sulfanilamide (C_6_H_8_N_2_O_2_S) as the standard, the ground fabrics were first burned, and the produced gases passed through a catalyst and a copper reduction stage, carried by helium gas, and then moved through a gas chromatography column. For oxygen determination, the system was operated in pyrolysis mode with aspartic acid (C_4_H_7_NO_4_) as the standard. The samples were weighed into silver containers and introduced into the pyrolysis reactor. Within the reactor, oxygen present in the sample reacted with carbon to produce carbon monoxide. This carbon monoxide was chromatographically separated from the other gaseous products. Finally, a thermal conductivity detector was used to detect all the gases and provide the elemental composition of the fabrics.^38,39^

X-ray photoelectron spectroscopy (XPS) measurements were performed using a Kratos AXIS Ultra DLD spectrometer equipped with a monochromated Al Kα X-ray source (1486.7 eV, 100 W). Survey and high-resolution spectra were collected under ultrahigh vacuum conditions (<1 × 10^–9^ Torr) using pass energies of 80 and 20 eV, respectively. Spectra were acquired from three different locations on each sample to assess surface homogeneity.^40^

FT-IR spectra of the dried fabric samples were obtained using a PerkinElmer FT-IR spectrometer equipped with an attenuated total reflectance (ATR) mode. The measurements were conducted within the spectral range of 4000–600 cm^–1^ at a resolution of 4 cm^–1^ with a background and sample scan time of 32 scans.^41^

Thermogravimetric analysis (TGA) was performed using a Netzsch STA 449 F3 Jupiter and QMS 403 Aëolos Quadro. Approximately 6–8 mg of each sample (undyed, dyed, and color-stripped fabrics) was placed in an Al_2_O_3_ crucible and analyzed under an N_2_ atmosphere (70 mL/min). The samples were heated from 40 to 600 °C at a heating rate of 10 °C/min.^42^

High-resolution images of all fabrics were captured to accurately represent their colors. A closed-chamber setup with a D65 light source and a Sony A7R3a camera equipped with an FE 90 mm F2.8 Sony macro lens was used to capture the images.^43^

A Shimadzu UV-1800 UV–vis spectrophotometer measured dye solution absorbance in the 200–700 nm range after 10-times dilution. The dye exhaustion (%), fixation efficiency (%), and fixation (%) were determined using eqs 7–9, respectively.

7 $$\mathrm{Exhaustion}\left(\%\right)=\left(1-\frac{A_{f}}{A_{i}}\right)\times 100$$

8 $${\mathrm{Fixation}}_{\mathrm{efficiency}}\left(\%\right)=\left(\frac{{\left(\frac{K}{S}\right)}_{\mathrm{a.s.}}}{{\left(\frac{K}{S}\right)}_{\mathrm{b.s.}}}\right)\times 100$$

9 $$\mathrm{Fixation}\left(\%\right)=\mathrm{Exhaustion}\left(\%\right)\times {\mathrm{Fixation}}_{\mathrm{eff}}\left(\%\right)$$

where A_i_ and A_f_ are the absorbance values of the dye solution before and after dyeing, respectively, and ${\left(\frac{K}{S}\right)}_{\mathrm{b.s.}}$ and ${\left(\frac{K}{S}\right)}_{\mathrm{a.s.}}$ represent the color strength of the dyed samples before and after soaping.

Color fastness to washing was evaluated according to ISO 105-C06-A1S:2010 using 4 g/L ECE reference detergent at 40 °C for 30 min with 10 steel balls. Rubbing fastness was evaluated following ISO 105-X12:2010, and color change and staining were assessed using SDC gray scales under a color-matching light cabinet.

All the fabrics were stored in an air-conditioned room before characterization, where the temperature was maintained at 23 ± 2 °C and the relative humidity at 66 ± 1%.

## 3 Results and Discussion

### 3.1 Process Optimization

On the basis of the literature and preliminary screening of experiments, chemical concentration, bath pH, temperature, reaction time, and material-to-liquor ratio were identified as key factors influencing the color stripping process.^44,45^ The optimization process was conducted to obtain the best output, considering lower chemical and energy usage in both the reductive and oxidative steps. These process parameters were optimized based on their corresponding color stripping % and L* values, which were considered independent response variables. DG_Dy_ was used to facilitate the optimization process. Figure 1 shows the process parameters, where a, b, and c represent the optimization of the concentration, pH, and temperature for the Na_2_S_2_O_4_-based reductive step, and d, e, and f illustrate the optimization of the H_2_O_2_-based oxidative step. The optimization of the reaction time and material-to-liquor ratio for both processes is mentioned in Figure S2.

**Figure 1:**
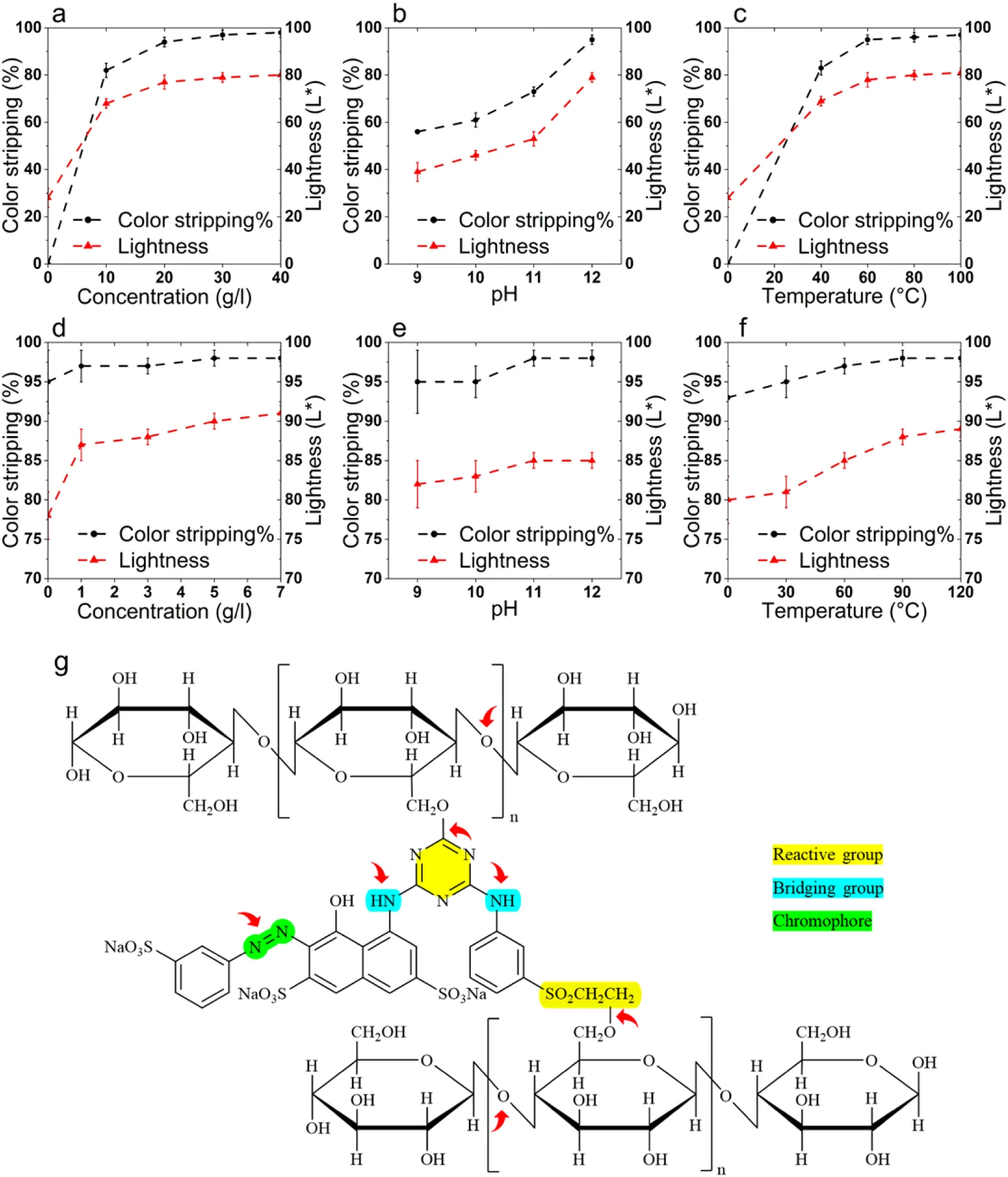
Optimization of a two-step reduction–oxidation bleaching process for dyed textile waste; (a–c) Reductive stage optimization by varying the Na_2_S_2_O_4_ concentration (a), pH (b), and temperature (c), the optimal reductive conditions were determined as 20 g/L Na_2_S_2_O_4_ at pH 12 and 60 °C for 60 min with an M:L ratio of 1:20; (d–f) Oxidative stage optimization by varying the H_2_O_2_ concentration (d), pH (e), and temperature (f), the optimal oxidative conditions were identified as 1 g/L H_2_O_2_ at pH 11 and 90 °C for 60 min with a material-to-liquor ratio of 1:20; (g) a schematic representation of the possible color-stripping mechanism and potential reaction sites (red arrows) in the dye-cellulose fiber linkage.

Color stripping increased from 82 ± 3 to 94 ± 2%, and L* increased from 28 ± 4 to 77 ± 3 with an increase in Na_2_S_2_O_4_ concentration from 10 to 20 g/L. A further increase to 30 g/L improved color stripping to 97 ± 2%, while at 40 g/L, the value reached 98 ± 2%, showing very limited improvement. However, the L* values increased by no more than 3 when the concentration was increased from 20 g/L to 40 g/L. Therefore, 20 g/L was considered the optimum concentration based on the color performance for subsequent treatments. Raising the bath pH from 9 to 12 significantly enhanced color removal, increasing from 56 ± 6% at pH 9 to 95 ± 2% at pH 12. Correspondingly, the L* improved from 39 ± 5 at pH 9 to 79 ± 2 at pH 12. The results showed that a strongly alkaline medium was necessary for effective reduction, and pH 12 was chosen as the optimum condition based on color removal and L*. When the temperature increased from 40 to 60 °C, color stripping increased from 83 ± 5% to 95 ± 4%, and L* improved from 69 ± 4 to 78 ± 3. A further increase to 80 °C showed 96 ± 3% color stripping and L* of 80 ± 5, whereas at 100 °C, the values reached 97 ± 2% and 81 ± 6. Therefore, 60 °C was selected as the optimum temperature. The optimal conditions for the reductive step were 20 g/L, pH 12, 60 °C, 60 min, and a material-to-liquor ratio of 1:20, which achieved approximately 95% color stripping and an L* value of around 79 while maintaining good resource efficiency, as presented in Figure 1(a–c).

Although the color stripping percentage was high, the fabric still appeared colored; therefore, L* could be a better indicator of whiteness. To further improve the L*, an oxidation process was introduced after the reduction. The optimization of this oxidation step was performed for all process parameters in the same manner as for the reduction. Increasing the H_2_O_2_ concentration up to 7 g/L produced a color stripping of 98 ± 1%, while the L* improved to 91 ± 1. The color stripping was the same at 1 and 3 g/L, and only slightly higher at 5–7 g/L (by about 1). The L* reached 87 at 1 g/L, which corresponded to a white appearance. The increase in L* at 3–7 g/L was marginal. Therefore, 1 g/L was considered the optimum concentration based on both color stripping and L* measurements. Likewise, pH 11, a temperature of 90 °C, and a reaction time of 60 min were chosen as the optimum conditions for oxidative treatment, as illustrated in Figure 1(d–f). Therefore, optimum oxidative treatment with H_2_O_2_ was achieved at a concentration of 1 g/L, pH 11, 90 °C, 60 min, and a liquor ratio of 1:20. Over 99% color-stripped fabric (DG_Rd‑Ox_) with above 89 L* was obtained using this optimized recipe.

### 3.2 Optimized Color Stripping Process

In the first step, Na_2_S_2_O_4_, a strong reducing agent, modifies the chromophores of the dyes, as evidenced by the changes in the absorption spectra (Table 3). However, it does not completely cleave the covalent bonds between the dye molecules and cellulose, which is confirmed by the partial retention of nitrogen and sulfur in the color-stripped fabrics (Table 6). In the subsequent oxidative step, hydrogen peroxide under alkaline conditions can effectively break the dye-cotton covalent linkages, as indicated by absence of further changes in the absorption spectra (Table 3). Consequently, the remaining color residues are removed, leading to a substantial improvement in the L* of the cotton fabric. The reactions of Na_2_S_2_O_4_ (1st step) and H_2_O_2_ (2nd step) are mentioned in eq 10 ^46^ and eq 11.^45^ A schematic representation of the possible stripping mechanism and potential reaction sites is depicted in Figure 1g.

10 $$2\;S_{2}{O_{4}}^{2-}+H_{2}O\to 2HS{O_{3}}^{-}+S_{2}{O_{3}}^{2-}$$

11 $$H_{2}O_{2}+H_{2}O⇄{\left(H_{3}O\right)}^{+}+H{O_{2}}^{-}$$

**Table 3:** Maximum Reflectance (*R*_max_), Maximum Wavelength (λ_max)_, K/S, Color Stripping (%), and ΔE_CMC_ of the Dyed, Reductive, and Reductive–Oxidative Stripped Fabrics

Fabrics	R_max_	λ_max_	K/S	Color stripping (%)	ΔE_CMC_ (compared with WH_Ud_)
**DG**_**Dy**_	3.8 ± 0.1	625	12.3 ± 0.5	-	26.1 ± 0.2
**DG**_**Rd**_	41.4 ± 0.1	410	0.4 ± 0.1	96.6 ± 0.4	12.0 ± 0.5
**DG**_**Rd‑Ox**_	65.4 ± 0.2	410	0.1 ± 0.1	99.2 ± 0.3	5.4 ± 0.3
**GR**_**Dy**_	8.7 ± 0.1	625	4.7 ± 0.3	-	20.2 ± 0.3
**GR**_**Rd**_	36.4 ± 0.2	410	0.5 ± 0.2	88.3 ± 0.2	12.2 ± 0.3
**GR**_**Rd‑Ox**_	45.2 ± 0.1	410	0.3 ± 0.1	93.1 ± 0.1	12.0 ± 0.1
**BL**_**Dy**_	1.9 ± 0.1	585	24.6 ± 0.3	-	27.8 ± 0.2
**BL**_**Rd**_	30.2 ± 0.2	410	0.8 ± 0.2	96.7 ± 0.2	9.0 ± 0.1
**BL**_**Rd‑Ox**_	56.7 ± 0.1	410	0.2 ± 0.1	99.3 ± 0.1	5.0 ± 0.1
**NV**_**Dy**_	2.5 ± 0.1	575	18.6 ± 0.3	-	27.2 ± 0.3
**NV**_**Rd**_	23.1 ± 0.2	410	1.2 ± 0.1	93.1 ± 0.4	21.6 ± 0.2
**NV**_**Rd‑Ox**_	46.4 ± 0.2	410	0.3 ± 0.1	98.3 ± 0.3	12.8 ± 0.2

### 3.3 Analysis of Colorimetric Properties

The colorimetric properties of the dyed and stripped fabrics are presented in Figure 2(a,b), Table 3, and Table S3. Following color stripping, the fabric reflectance increased, while the absorption coefficient (K) decreased and the scattering coefficient (S) increased, indicating the destruction of dye chromophores and the resulting lighter appearance of the fabrics (Table 3 and Table 4). The K/S values dropped significantly after reduction and decreased further after oxidation (Figure S3). Among the four fabrics, the black sample (BL_Dy_) responded most effectively to the reductive process, achieving 96.7 ± 0.2% color stripping, and reached the highest efficiency (99.3 ± 0.1%) after the sequential reduction–oxidation treatment. Similarly, the sequential treatment produced 99.2 ± 0.3% color stripping for DG_Rd‑Ox_, 98.3 ± 0.3% for NV_Rd‑Ox_, and 93.1 ± 0.1% for GR_Rd‑Ox_. In addition, the consistent shift of λ_max_ toward 410 nm confirmed the removal of long-wavelength dye chromophores; the subsequent oxidation step eliminated the residual dye fragments left after reduction, raising the overall color stripping efficiency to ≥ 99%.

**Figure 2:**
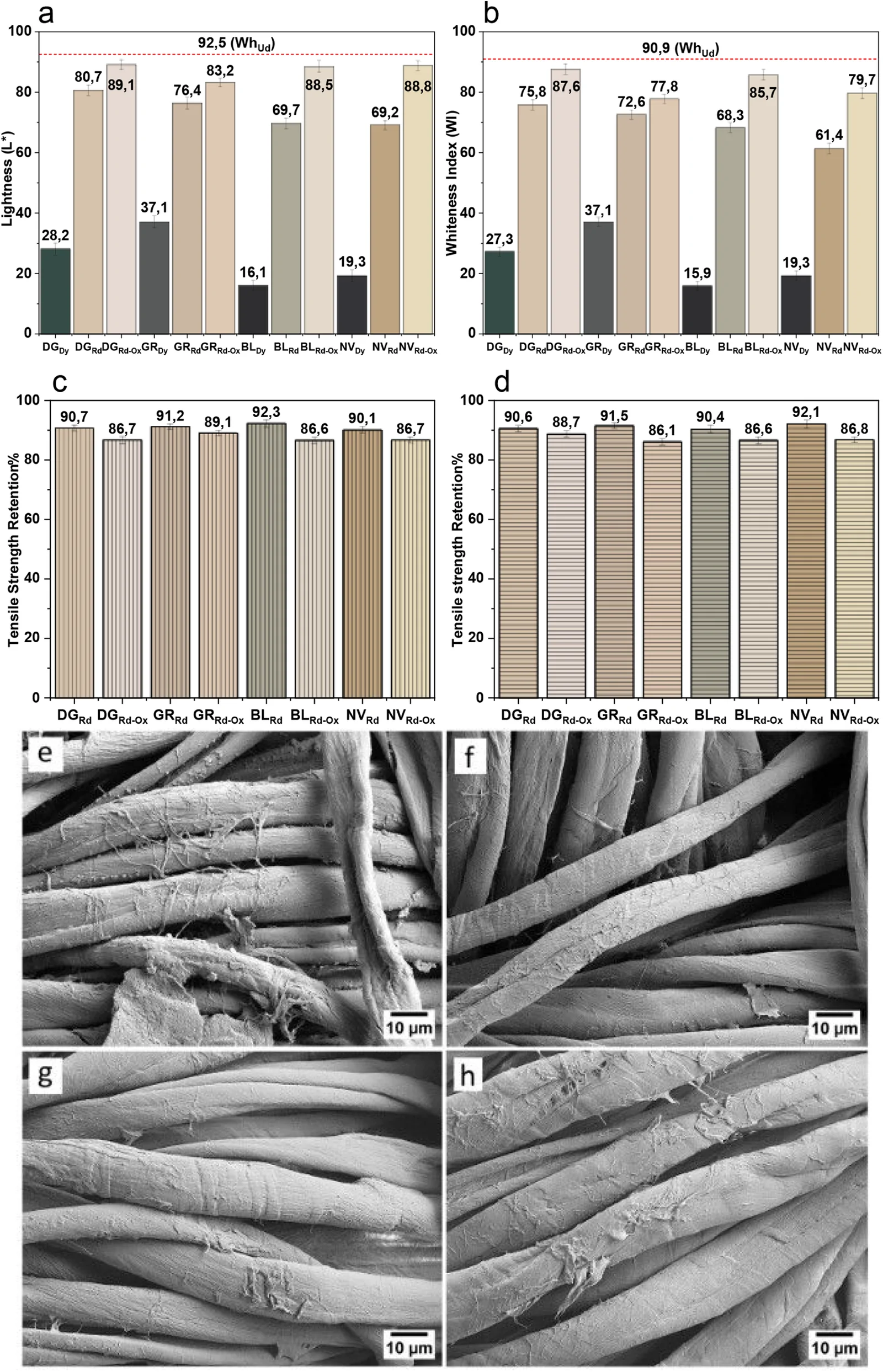
(a) L*, (b) WI of the dyed and color-stripped fabrics, tensile strength retention% of the color-stripped fabrics, representing (c) warp, (d) weft orientation, and SEM images of (e) WH_Ud_, (f) BL_Dy_, (g) BL_Rd_, and (h) BL_Rd‑Ox_ fabrics.

**Table 4:**
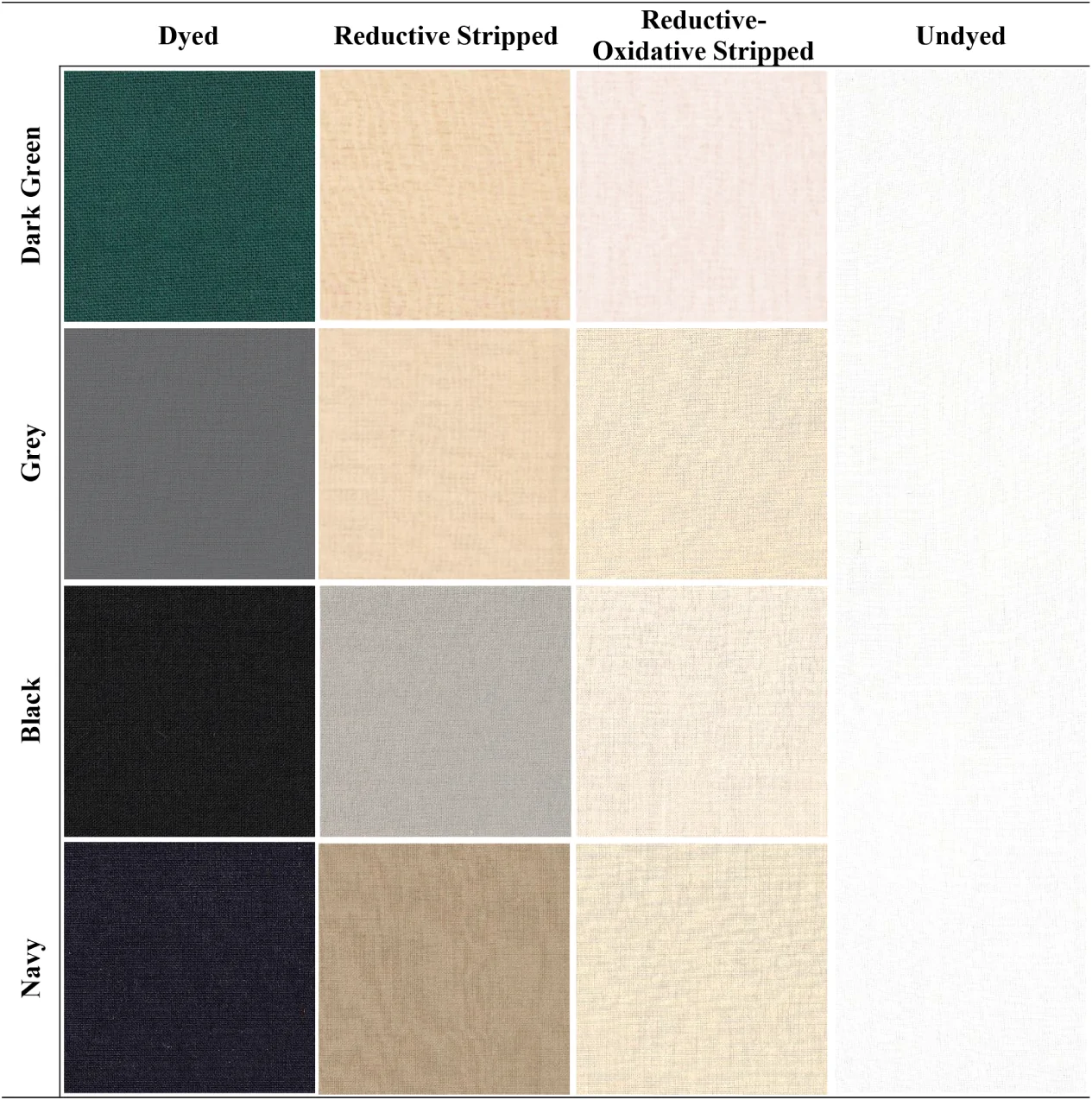
Images of the Dyed and Color-Stripped Fabrics Compared with the Undyed Fabric

Using the WH_Ud_ as a reference, the ΔE_CMC_ decreased significantly from the dyed state to the reductive–oxidative state. In comparison to the selected white standard, ΔE_CMC_ was ≥5 for both DG_Rd‑Ox_ and BL_Rd‑Ox_, indicating the dye removal and their substantial K/S value drop (Table 3). In contrast, GR_Rd_ and GR_Rd‑Ox_ showed less variation from reduction (12.2 ± 0.3) to oxidation (12 ± 0.1), and NV_Rd‑Ox_ remained at 12.8 ± 0.2, despite having a very low K/S value (0.3 ± 0.1). This difference is likely because ΔE_CMC_ represents perceptual color difference and is influenced by residual dye fragments; the persistent wavelength of 410 nm left by the leftover dye fragments elevated ΔE_CMC_ even when K/S was low.

As shown in Figure 2a, all dyed fabrics exhibited much lower L* values than the undyed reference (WH_Ud_, 92.5 ± 1.5) due to strong visible light absorption by chromophores. After the reduction step, L* increased markedly across all samples, indicating substantial chromophore destruction. The subsequent oxidation step further enhanced L*, with DG_Rd‑Ox_ and NV_Rd‑Ox_ reaching values closest to WH_Ud_. All stripped fabrics exhibited L* values above 80, which suggests that the fabrics achieved a noticeably light appearance. Moreover, the redness (a*) and blueness (b*) are reported in Figure S4 and Table S3 by presenting the CIE L*a*b* space of undyed, dyed, and color-stripped fabrics. A similar trend to L* was observed for the WI. After the reduction step, the WI improved to 61.4 – 75.8, as most of the chromophores were removed. The subsequent oxidation step further increased the WI to 77.8 – 85.7 by eliminating the remaining residual chromophores. DG_Rd‑Ox_ and BL_Rd‑Ox_ thus approached the WH_Ud_ (WI = 90.9 ± 1), as shown in Figure 2b.

The images of the dyed and color-stripped fabrics, compared with the WH_Ud_ are shown in Table 4, and the corresponding images from the X-Rite reflectance spectrophotometer are presented in Table S5.

### 3.4 Analysis of Mechanical Properties

The effects of reductive and oxidative color-stripping treatments on the tensile properties of the dyed and color-stripped fabrics are presented in Figure 2(c,d) as tensile strength retention (%), while the corresponding tensile strength (MPa) and elongation at break (%) values in both the warp and weft directions are summarized in Table S4. For the dark green fabric, a 9.3% strength loss was observed in the warp direction after reduction, with an additional 4% loss after oxidation. In the weft direction, the corresponding losses were 9.4% after reduction and a further 1.9% after oxidation. A similar trend was observed for the other fabrics: GR, BL, and NV. Therefore, the tensile strength retention% in the warp direction was 86.7% for DG_Rd‑Ox_, 89.1% for GR_Rd‑Ox_, 86.6% for BL_Rd‑Ox_, and 86.7% for NV_Rd‑Ox_. In the weft direction, the corresponding values were 88.7%, 86.1%, 86.6%, and 86.8% for DG_Rd‑Ox_, GR_Rd‑Ox_, BL_Rd‑Ox_, and NV_Rd‑Ox_, respectively. A moderate tensile strength loss was observed after Na_2_S_2_O_4_ treatment because the reducing agent affected not only the dye chromophores but also weakened the cotton structure. In the subsequent step, H_2_O_2_ cleaved the residual dye–cellulose covalent bonds; however, the extent of fiber damage was mitigated by the stabilizer present in the H_2_O_2_ solution.^18^ Overall, the tensile strength retention% of all the fabrics was in the range of 86 – 92%, which confirmed the compatibility of the color-stripped fabrics with recycling.

The color-stripping treatments also influenced the weight loss of dyed fabrics, as presented in Table 5. During the reductive step, weight loss ranged from 1.3 ± 0.1% to 2.7 ± 0.3% across all fabrics, with the minimum and maximum values observed for GR_Rd_ and BL_Rd_, respectively. An additional weight loss of about ±1% was consistently recorded after oxidation for all fabric variants. Following the complete reductive–oxidative sequence, the total weight loss reached 2.5 ± 0.4% for DG_Rd‑Ox_, 2.2 ± 0.3% for GR_Rd‑Ox_, 3.5 ± 0.5% for BL_Rd‑Ox_, and 3.2 ± 0.3% for NV_Rd‑Ox_. The higher weight loss observed during reduction is attributed to the removal of the bulk of dye molecules, while the subsequent oxidation contributed less since only residual dyes were removed. Furthermore, variations in the overall weight loss among different fabrics may be related to the different dye loadings applied during the dyeing process (Table 1).

**Table 5:** Weight Loss (%) and Degree of Polymerization of the Reductive and Reductive-Oxidative Color-Stripped Fabrics

Fabrics	Weight loss (%)	Degree of polymerization
**WH**_**Ud**_	-	3395 ± 29
**DG**_**Rd**_	1.7 ± 0.2	2144 ± 21
**DG**_**Rd‑Ox**_	2.5 ± 0.4	1495 ± 18
**GR**_**Rd**_	1.3 ± 0.1	2073 ± 25
**GR**_**Rd‑Ox**_	2.2 ± 0.3	1404 ± 19
**BL**_**Rd**_	2.7 ± 0.3	2250 ± 27
**BL**_**Rd‑Ox**_	3.5 ± 0.5	1701 ± 20
**NV**_**Rd**_	2.4 ± 0.2	2181 ± 22
**NV**_**Rd‑Ox**_	3.2 ± 0.3	1576 ± 14

### 3.5 Analysis of Morphological Properties

SEM images of undyed, dyed, and stripped fabrics are shown in Figure 2(e–h). The undyed fabric exhibited the characteristic twisted and flattened ribbon-like morphology of cotton fibers (Figure 2e). In contrast, the dyed fabric displayed a cleaner and smoother surface due to the deposition of dyes and auxiliaries, along with the appearance of shallow microcracks on the fiber surface (Figure 2f). After reduction treatment, the fabric surface became rougher (Figure 2g), which may be attributed to the removal of the dye chromophores attached to the cotton surface, as Na_2_S_2_O_4_ primarily attacks the dye chromophoric groups rather than the dye–fiber covalent bonds.^18^ In comparison, oxidation treatment with H_2_O_2_ targeted the dye–fiber covalent bonds, resulting in comparatively rougher surfaces and localized fibrillation (Figure 2h). These SEM observations are consistent with the measured higher tensile strength retention% and lower weight loss values of the stripped fabrics.

Figure 3(a–d) depicts the XRD pattern of undyed, dyed, and color-stripped cotton fabrics. The fabrics exhibit the expected cellulose I pattern, characterized by the diffraction peaks (1–10), (110), (200), and (004), observed at 15°, 16°, 22°, and 34°, respectively.^47^ WH_Ud_ possesses a crystallinity index (CI) of 49% (Table S6), which is consistent with previous studies on cotton roll towels (41–44%).^48^ Dyeing showed almost no effect on the crystallinity index. Reductive, as well as reductive–oxidative treatments, cause the loss of amorphous regions, resulting in a slight increase of CI, reaching a maximum value of 53%. However, the impact of the treatment appears to be minimal.^48–50^ All samples exhibit a similar trend, as detailed in Figure 3(a–d).

**Figure 3:**
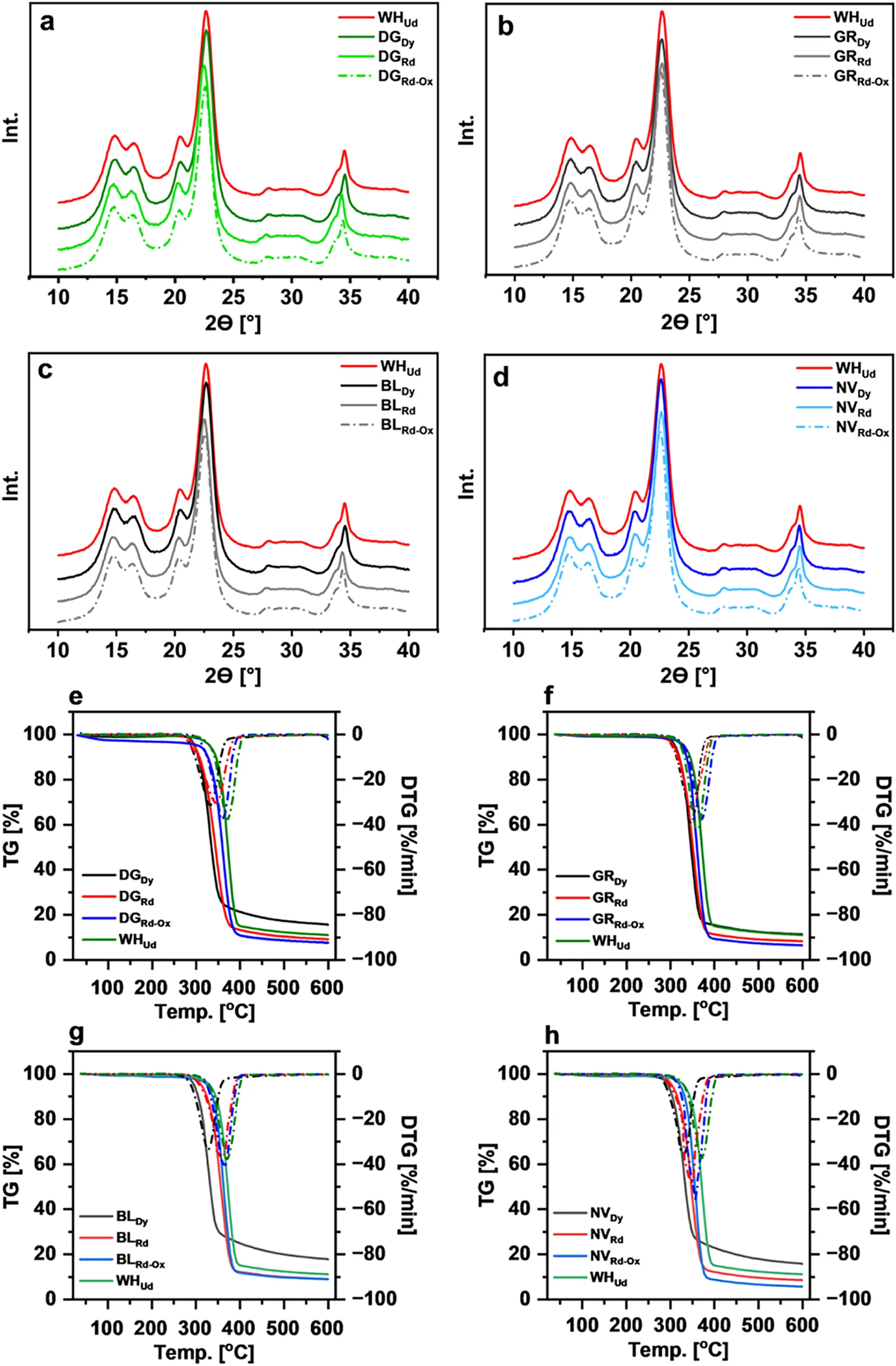
XRD plots of dyed, reductive, and reductive-oxidative stripped fabrics of (a) DG, (b) GR, (c) BL, and (d) NV compared to WH_Ud_ and the thermal profile of dyed, reductive, and reductive-oxidative stripped fabrics of (e) DG, (f) GR, (g) BL, and (h) NV compared to WH_Ud_ illustrated via TG (%) and DTG (%/min) vs Temperature (°C).

### 3.6 Analysis of Intrinsic Viscosity and Degree of Polymerization

The intrinsic viscosity and degree of polymerization for color-stripped fabrics are presented in Table S7 and Table 5, respectively. The DP of the undyed cotton fabric (WH_Ud_) was 3395 ± 29 (intrinsic viscosity: 1099 ± 10). After reduction treatment, the DP decreased to 2144 ± 21 for DG_Rd_, 2073 ± 25 for GR_Rd_, 2250 ± 27 for BL_Rd_, and 2181 ± 22 for NV_Rd_. This indicates that Na_2_S_2_O_4_ did not extensively affect the cellulose chain, but primarily targeted dye chromophores. Following oxidation with H_2_O_2_, the DP values decreased further, ranging from 1404 ± 19 to 1701 ± 20 across the color-stripped fabrics. This additional loss reflects partial depolymerization under stabilized alkaline H_2_O_2_, while also accounting for the removal of residual dyes.^51^ Overall, the DP changed from 3395 ± 29 (undyed) to 1404–1701 (after reductive–oxidative treatment). The decrease in DP may be due to the preferential breakdown of cellulose chains. Both the amorphous and crystalline regions are affected, as illustrated by a minor change in crystallinity (Table S6). As a result, the ratio of crystalline to amorphous regions stayed nearly unchanged, and the organized fibrillar structure, which primarily maintains higher tensile strength, remained similar.^48,49^ The achieved DP of 1400 to 1600 aligns with the values commonly found for dissolving pulp used for fiber manufacturing. In cellulose pulp processing, lignin, hemicelluloses, and other extractable substances are removed as much as possible. This is followed by controlled degradation to produce cellulose with a DP of 952 to 1429, corresponding to an intrinsic viscosity of 400 to 600 mL/g.^52–54^ Asaadi et al. state that the intrinsic viscosity of cellulose greatly influences its processability during chemical recycling with the dry-jet wet fiber spinning method.^55^ Good spinnability was observed with materials having an intrinsic viscosity of 400–500 mL/g, corresponding to a DP of 1000–1200. Besides, the recyclability of cotton into high-quality fibers has been demonstrated, enabling further use of the decolorized materials from this research.^48,55,56^

### 3.7 Analysis of Bulk and Surface Elemental Composition

Cotton fiber is composed of about 90–95% cellulose, a carbohydrate consisting of carbon (44.4%), hydrogen (6.2%), and oxygen (49.4%), with its molecular structure built from repeating units of cellobiose.^57^ In contrast, dyed fabrics contain additional heteroatoms such as nitrogen (N) and sulfur (S), introduced during the dyeing process (Table S2). The elemental compositions of undyed, dyed, and color-stripped fabrics are presented in Table 6. It is evident that the amounts of S and N decreased after the reductive treatment, which can be attributed to the removal of dye chromophores from cotton. After oxidative treatment, only a negligible trace of nitrogen (0.1%) and no detectable sulfur remained, suggesting the breakage of dye–fiber covalent bonds. The findings of the reduction–oxidation treatment confirmed that all fabric variants returned to the elemental composition of WH_Ud_, indicating that the dyes were successfully stripped. The proportions of oxygen (≈48.1%), carbon (≈35.1%), and hydrogen (≈5.8%) were restored to levels comparable to those of the undyed fabric. This compositional reversion from dyed to color-stripped fabrics is consistent with the observed colorimetric properties, including reduced color strength, increased L*, and a higher WI.

**Table 6:** Changes in Elemental Composition of Undyed, Dyed, and Color-Stripped Fabrics^a^

	Elements (%)				
Samples	Oxygen	Carbon	Hydrogen	Nitrogen	Sulfur
**WH**_**Ud**_	48.3 ± 0.1	35 ± 0.1	5.8 ± 0.3	0.1 ± 0.02	ND
**DG**_**Dy**_	48.6 ± 0.2	43 ± 0.3	6.2 ± 0.1	0.3 ± 0.02	0.2 ± 0.01
**DG**_**Rd**_	48.6 ± 0.3	42.9 ± 0.3	6.1 ± 0.2	0.2 ± 0.04	0.1 ± 0.01
**DG**_**Rd‑Ox**_	48.1 ± 0.3	35.1 ± 0.5	5.8 ± 0.4	0.1 ± 0.03	ND
**GR**_**Dy**_	48.5 ± 0.2	43.2 ± 0.2	6.3 ± 0.3	0.2 ± 0.03	0.1 ± 0.01
**GR**_**Rd**_	48.5 ± 0.2	42.9 ± 0.1	6.2 ± 0.3	0.2 ± 0.01	0.1 ± 0.01
**GR**_**Rd‑Ox**_	48.1 ± 0.3	35.1 ± 0.3	5.8 ± 0.2	0.1 ± 0.02	ND
**BL**_**Dy**_	48.6 ± 0.5	43.9 ± 0.4	6.2 ± 0.5	0.4 ± 0.02	0.5 ± 0.03
**BL**_**Rd**_	48.5 ± 0.4	42.9 ± 0.1	5.8 ± 0.2	0.2 ± 0.03	0.2 ± 0.01
**BL**_**Rd‑Ox**_	48.1 ± 0.2	35.2 ± 0.2	5.8 ± 0.4	0.1 ± 0.03	ND
**NV**_**Dy**_	48.5 ± 0.2	43.9 ± 0.4	6.2 ± 0.5	0.4 ± 0.01	0.3 ± 0.01
**NV**_**Rd**_	48.4 ± 0.4	42.9 ± 0.1	5.8 ± 0.3	0.2 ± 0.02	0.2 ± 0.02
**NV**_**Rd‑Ox**_	48.1 ± 0.2	35 ± 0.3	5.8 ± 0.3	0.1 ± 0.03	ND

a [ND: not detected (below detection limit of <100 ppm).].

As shown in Table S9, XPS analysis revealed significant changes in the surface elemental composition after color stripping. The carbon content decreased from 76.36% to 68.45%, while the oxygen content increased from 21.79% to 31.36%, which may indicate the formation of additional oxygen-containing functionalities on the cellulose surface due to oxidative treatment. Notably, the elemental composition of the treated sample approached that of the untreated cotton reference, where 65.1% C and 34.9% O,^58^ suggesting partial restoration of the native cellulose surface chemistry. In addition, sulfur and nitrogen contents decreased markedly from 0.52% to 0.02% and from 0.51% to 0.12%, respectively, confirming the effective removal of dye-derived species. The O/C atomic ratio increased from 0.29 (dyed fabric) to 0.46 (color-stripped fabric), approaching the value of 0.5 for untreated cotton,^59^ further demonstrating that the treated surface became chemically more similar to native cellulose following color stripping. These XPS results are in good agreement with the bulk elemental analysis results in Table 6, further confirming the effective removal of dye-related elements and the efficient decolorization of the textile waste.

These changes in elemental composition were further supported by FT-IR (ATR) analysis of the dyed and color-stripped fabrics, with the corresponding spectra presented in Figure S5.

### 3.8 Analysis of Thermal Characteristics

The thermal characteristics of undyed, dyed, and color-stripped fabrics are presented in Figure 3(e–h) and Table S8. All fabrics exhibited similar thermal behavior. The dyed fabrics showed slightly lower onset degradation temperatures (T_onset_) than the undyed fabrics, indicating that the presence of dyes reduces the thermal stability of cotton. Although bifunctional reactive dyes can form covalent bonds with cellulose and cause limited cross-linking, this interaction does not necessarily improve thermal resistance. The attached dye molecules contain heteroatoms and thermally unstable substituents that may trigger early decomposition. Also, dyes present in the fabrics reduce the overall mass loss, leaving more residue at 600 °C. After dye removal through reductive and sequential reductive–oxidative treatments, the onset degradation temperatures increased, and the thermal profiles of the color-stripped fabrics approached those of the undyed cotton, aligning well with values reported in the literature.^48,60^

### 3.9 Analysis of Redyeability Performance

The images of the redyed fabrics from the X-Rite Spectrophotometer, K/S, ΔE_CMC_, E%, F%, and color fastness properties of the redyed fabrics are evaluated and presented in Table 7. The redyed fabrics exhibited color strengths comparable to the dyed_Reference_ fabric, indicating effective dye uptake after the reductive–oxidative stripping treatment. The K/S values of the redyed fabrics ranged from 2.1 ± 0.2 to 2.8 ± 0.1, showing only minor variation compared to the reference (2.8 ± 0.1). The corresponding ΔE_CMC_ values between the redyed and reference fabrics were found to be ≤1.8, suggesting that the redyed shades were close to the reference dyed fabric. Dye exhaustion (72–79%) and fixation (66–77%) exhibited high values for all redyed fabrics, confirming that the color-stripped fabrics retained good dyeing performance. Among all, the BL_Rd–Ox_ fabric achieved the highest exhaustion (79.3%) and fixation (76.5%), almost identical to the reference. All redyed fabrics exhibited excellent washing and rubbing fastness (4–5 to 5), comparable to the dyed_Reference_. Moreover, CIE L*a*b* space and F_eff_% are mentioned in Table S10. These results confirmed that color-stripped fabrics showed excellent redyeability properties.

**Table 7:**
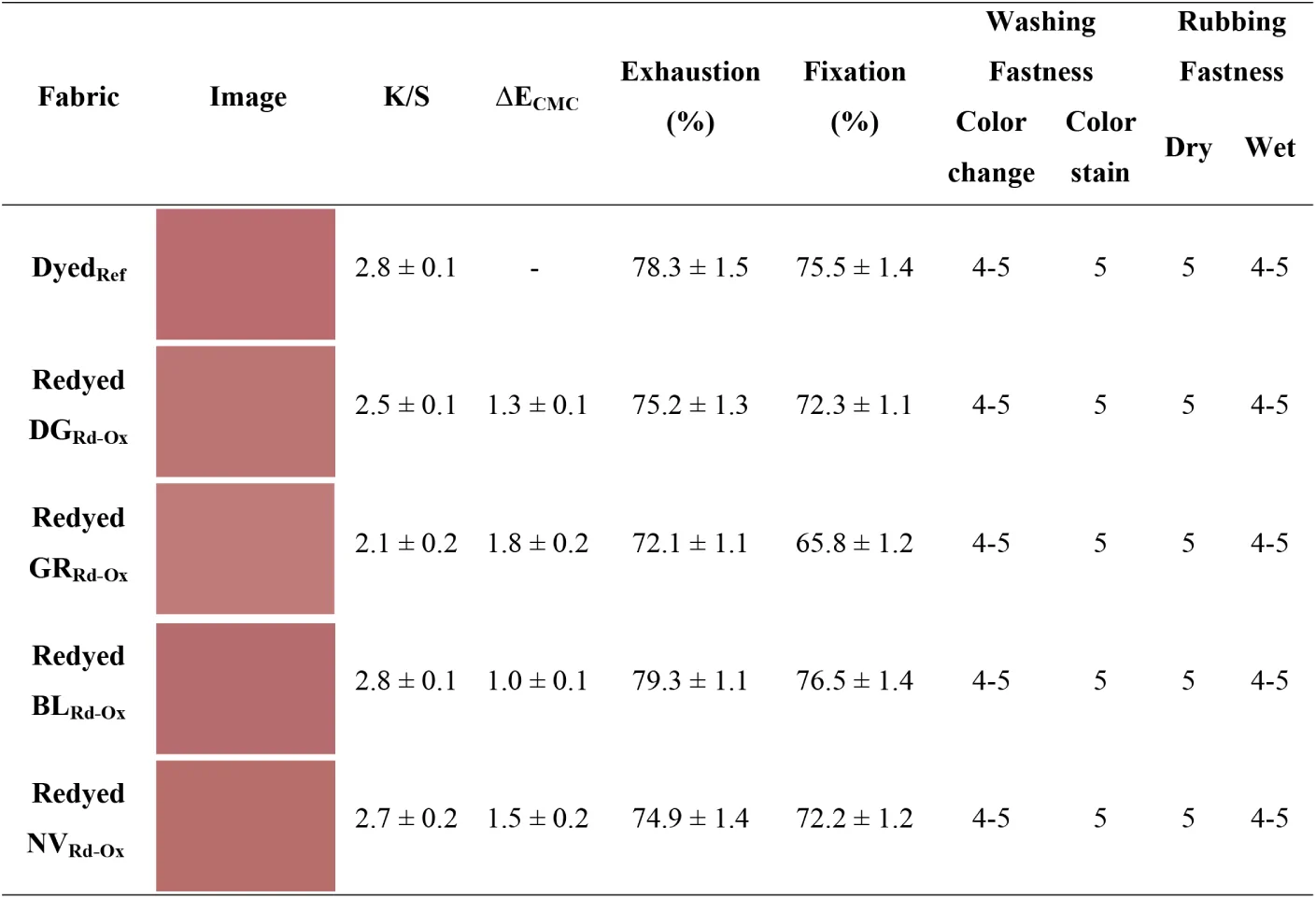
Dyed_Reference_ and Redyed Fabric Images, K/S, ΔE_CMC_, Exhaustion (%), Fixation (%), Washing Fastness, and Rubbing Fastness Scores

## 4 Conclusions

We developed a sequential color-stripping strategy to tackle the challenges of reactive dyes from cotton waste. The colorimetric, mechanical, thermal, morphological, elemental, and polymeric properties of cotton fabrics were evaluated to explain the effects of this sequential treatment. Dark Green, Gray, Black, and Navy cotton fabrics dyed with mixed reactive dyes were effectively decolorized. The findings revealed that the optimized color-stripping process resulted in 93–99% color removal, with L* ranging from 83 to 89. The WI of the treated fabrics (77–87) indicates that the color-stripped cotton can be successfully reused for redyeing and chemical recycling. The effect of the sequential process on cotton was achieved with a minimal effect on the mechanical properties, and the average tensile strength retention% was above 86% in both warp and weft orientations, while weight preservation was over 96%. Mild fibrillation and a slight increase in crystallinity confirm the integrity of the cotton morphology. The intrinsic viscosity was about 550–650 mL/g, and the DP was about 1400–1700, which is close to the range of materials used for chemical recycling. Furthermore, the color-stripped fabrics exhibited elemental compositions by XPS and CHNSO analyses and thermal degradation behavior from TGA, nearly identical to those of the undyed cotton, providing strong evidence for the successful removal of dyes from the cotton waste. Moreover, color-stripped fabrics showed excellent redyeability properties (ΔE_CMC_ ≤1.8; color fastness: 4–5 to 5).

Although the present work employs a batch methodology, the process can be conceptually extended to continuous operation using established textile processing systems such as pad–batch or continuous washing ranges. The sequential reduction–oxidation steps may be configured as consecutive treatment zones with controlled residence time and intermediate rinsing. Such configurations would enable integration with existing industrial infrastructure and facilitate scale-up, although detailed process design remains a subject for future investigation. This sequential color-stripping strategy is highly effective for removing covalently bonded reactive dyes from cotton fabrics. The two-step reduction–oxidation process successfully preserves the mechanical strength and morphological integrity of the fibers. Despite the promising decolorization performance and preservation of fiber properties, further investigations are required to address several process-scale challenges, including water and energy consumption, effluent management, chemical recovery, continuous process development, and the implementation of deoxygenated conditions to enhance process safety and further minimize cellulose degradation. These limitations should be carefully addressed before large-scale industrial implementation can be considered. The aforementioned aspects are among the key focuses of our ongoing and future research, and our research group is actively working to evaluate and optimize these factors through dedicated techno-economic, environmental, and scale-up studies.

From a sustainability perspective, this approach utilizes process chemicals that are already widely employed in textile- and pulp-related industries, potentially facilitating industrial implementation. While alternative technologies, such as enzymatic, plasma, or biological treatments, have also been explored for dye removal, these methods may involve higher operational complexity or limitations when applied to strongly fixed reactive dyes on textile substrates. In this context, the proposed process may offer a practically accessible route for textile-to-textile recycling, particularly when integrated with existing chemical recovery infrastructure in the pulp industry. Future work will focus on valorizing the color-stripped cotton by mechanical and chemical recycling, thereby contributing to a sustainable closed-loop recycling pathway for the textile industry.

## Supplementary Material



## Data Availability

The data presented herein are consistent with this article.

## Notes

Tables S1–S10: Comparative performance evaluation of reported color-stripping methods for reactive-dyed cotton textile waste; reactive dye characteristics and chemical structures; additional colorimetric parameters; tensile strength and elongation properties; fabric images; crystallinity index; intrinsic viscosity; thermal degradation and mass-loss data; XPS surface elemental composition; and CIE Lab* values and fixation efficiency of redyed samples. Figures S1–S5: Stepwise reductive–oxidative color-stripping process; screening of process parameters; K/S versus wavelength spectra; CIE Lab* color space plots; and FT-IR spectra of dyed, color-stripped, and undyed fabrics. (PDF)

## References

[1] EEA.Circularity of the EU textiles value chain in numbers, 2025. https://www.eea.europa.eu/en/analysis/publications/circularity-of-the-eu-textiles-value-chain-in-numbers (accessed 15 September 2025).

[2] Rohan, S. ; Catharina, M. P. ; Alexander, M. F. ; Tom, L. ; Eleonora, T. ; Elian, E. Spinning Textile Waste into Value; Boston Consulting Group, 2025. https://www.bcg.com/publications/2025/spinning-textile-waste-into-value (accessed 10 October 2025).

[3] Emam H. E., Koto T., Sebok-Nagy K., El-Shahat M., Abdel-Gawad H., Pali T., Abdelhameed R. M. (2024). Synthesis,
spectroscopic study and carbofuran adsorption of mixed metal (Co,
Cu)@Ca-BTC frameworks aimed at wastewater cleaning. J. Ind. Eng. Chem..

[4] Ahmed H. B., Saad N., Emam H. E. (2021). Recyclable palladium
based nano-catalytic
laborer encaged within bio-granules for dye degradation. Surf. Interfaces.

[5] Walawska A., Olak-Kucharczyk M., Kaczmarek A., Kudzin M. H. (2024). Environmentally
Friendly Bleaching Process of the Cellulose Fibres Materials Using
Ozone and Hydrogen Peroxide in the Gas Phase. Materials.

[6] Meurs E., Morshed M. N., Kahoush M., Kadi N. (2024). Study on Fenton-based
discoloration of reactive-dyed waste cotton prior to textile recycling. Sci. Rep..

[7] Zulfiqar A., Arooj F., Aftab M., Rashid M., Luqman M., Kashif S. U. R., Naseer R. (2023). A Sustainable Approach
to Dyed Cotton
Fabric Stripping Using Ozone. Sustainability-Basel.

[8] Atav R., Yüksel M. F., Özkaya S., Bugdayci B. (2022). The Use of Ozone Technology
for Color Stripping and Obtaining Vintage Effect on Reactive Dyed
Cotton Fabrics. J. Nat. Fibers.

[9] Powar A., Perwuelz A., Behary N., Hoang L. V., Aussenac T., Loghin C., Maier S. S., Guan J. P., Chen G. Q. (2021). Investigation
into the color stripping of the pigment printed cotton fabric using
the ozone assisted process: A study on the decolorization and characterization. J. Eng. Fibers Fabr..

[10] Powar A., Perwuelz A., Behary N., Hoang L., Aussenac T., Loghin C., Maier S. S., Guan J. P., Chen G. Q. (2021). Environmental
Profile Study of Ozone Decolorization of Reactive Dyed Cotton Textiles
by Utilizing Life Cycle Assessment. Sustainability-Basel.

[11] Arooj F., Ahmed N., Shaikh I. A. (2020). Application
of Ozone in Stripping
of Cotton Fabric Dyed with Reactive Dyes. Ozone-Sci.
Eng..

[12] Kicik H., Eren H. A. (2017). Application of ozone gas for the
stripping of fabric
ink-jet-printed with reactive dyes. Color. Technol..

[13] Song R., Fu L., Liu Z., Zhu K., Zhang M., Zhang J., Zeng B., Wang J. (2025). Closed-loop
recycling for colored
cotton textile waste. Sustainable Mater. Technol..

[14] Pervin M., Sultana S., Fahmi F. F., Yasmin Z., Swati S. S. (2022). Evaluation
of the Stripping Performance of Monochlorotriazine/Vinyl Sulphone
Reactive Dyes with a Reductive Stripping Agent. Tekstilec.

[15] Atav R., Çagman F. N., Sahin H., Çolakoglu Ö. (2022). Determination
of Color Removal and Fading Performance of Environmentally Friendly
Chemical Alternatives to Hydrosulfite in Reactive Dyed Cotton Knitted
Fabrics Depending on Dye Chromophore. J. Nat.
Fibers.

[16] He F. N., Li X., Zhu M. K., Hu J. H., Yuan Y. J., Li C. C., Long J. J. (2019). Color stripping
of reactive-dyed cotton fabric in a
UV/sodium hydrosulfite system with a dipping manner at low temperature. Cellulose.

[17] Uddin M. G., Islam M. M., Islam M. R. (2015). Effects
of reductive stripping of
reactive dyes on the quality of cotton fabric. Fash. Text..

[18] Li R., Yang J., Zhang G., Zhu P. (2022). Decolorization of dark-colored
waste cotton fabric using redox decoloring agents. RSC Adv..

[19] Bigambo P., Carr C. M., Sumner M., Rigout M. (2021). Investigation into
the removal of pigment, sulphur and vat colourants from cotton textiles
and implications for waste cellulosic recycling. Color. Technol..

[20] Määttänen M., Asikainen S., Kamppuri T., Ilen E., Niinimäki K., Tanttu M., Harlin A. (2019). Colour management in circular economy:
decolourization of cotton waste. Res. J. Text
Appar..

[21] Saha T., Le U., Repon M. R., Schlapp-Hackl I., Vuorinen T., Tehrani-Bagha A. (2026). An effective
color stripping process for reactive dyed cotton waste. Ind. Crop. Prod..

[22] Latif Z., Shafique J., Summuna B., Lone B., Ur Rehman M., El-Sheikh M. A., Hashim M. J., Vladulescu C., Shafique T. (2021). Development of efficient strain of Ganoderma lucidum
for biological stripping of cotton fabric dyed Reactive Blue 21. Saudi J. Biol. Sci..

[23] Chatha S. A. S., Asgher M., Ali S., Hussain A. I. (2012). Biological color
stripping: A novel technology for removal of dye from cellulose fibers. Carbohydr. Polym..

[24] Mariam M., Noureen S., Ali S., Khalid U. (2024). Unravelling the role
of microwave absorber in color stripping of a dyed natural polymeric
material with microwave assisted method. Cellulose.

[25] Younas T., Tayyaba N., Ali S. (2025). Insights into microwave-assisted
stripping of reactive black 5: a comparative perspective. Cellulose.

[26] Akhtar A., Alam M., Noureen S., Ali S., Tahir A. T., Shoukat A. (2024). Transforming waste cellulosic fabric
dyed with Reactive
Yellow C4GL into value textile by a microwave assisted energy efficient
system of color stripping. Heliyon.

[27] Alam M., Ali S., Noureen S., Zahira T., Akhtar A., Khan M. A. A. (2023). Study
of microwave-assisted sequential color stripping of cellulosic fabric
dyed with reactive blue black 5 and reactive turquoise CLB. Cellulose.

[28] Patil H., Panda A., Maiti S., Athalye A., Adivarekar R. V. (2024). Solvent-based
stripping method for dyed cellulosic textiles. J. TEXT I.

[29] Gellerstedt, G. Chemistry of Bleaching of Chemical Pulp. In Pulping Chemistry and Technology; De Gruyter, 2009; Vol, 2; pp. 391. 10.1515/9783110213423

[30] Gellerstedt, G. Pulping Chemistry and Technology, Volume 2; De Gruyter, 2009; Vol, 2; pp. 201-237 10.1515/9783110213423.201.

[31] Peter, W. H. Pulp Bleaching. In Kirk-Othmer Encyclopedia of Chemical Technology; John Wiley & Sons, Inc., 2019; pp. 1–30 10.1002/0471238961.1621121613030415.a01.pub3.

[32] Suess, H. U. Pulp Bleaching Today; De Gruyter, 2010. 10.1515/9783110218244

[33] Bigambo P., Carr C. M., Sumner M., Rigout M. (2020). The effect of the acid/dithionite/peroxide
treatments on reactively dyed cotton and indigo dyed denim and the
implications for waste cellulosic recycling. J. Text. Inst..

[34] ISO Domestic Washing And Drying Procedures For Textile Testing (ISO 6330: 2021), 2021. https://www.iso.org/obp/ui/#iso:std:iso:6330:ed-4:v1:en.

[35] Roderick, M. Colour physics for industry, edited by Roderick McDonald; Society of Dyers and Colourists, 1987 10.1002/col.5080130412.

[36] Chamseddine G., Sauli L., Kaniz M., Daisuke S., Sherif E., Sami R., Michael H., Herbert S. (2021). Air gap spinning of
a cellulose solution in [DBNH]­[OAc]­ionic liquid with a novel vertically
arranged spinning bath to simulate a closed loop operation in the
Ioncell® process. J. Appl. Polym. Sci..

[37] Abdi B., Baniasadi H., Tarhini A., Tehrani-Bagha A. (2025). Enhancing
Electrical Conductivity in Cellulosic Fabric: A Study of Bio-Based
Coating Formulations. Adv. Mater. Technol..

[38] Blas-Sevillano R. H., Veramendi T., La Torre B., Velezmoro-Sánchez C. E., Oliva A. I., Mena-Martínez M. E., Herrera-Kao W. A., Uribe-Calderon J., Cervantes-Uc J. M. (2018). Physicochemical characterization
of several types of naturally colored cotton fibers from Peru. Carbohydr. Polym..

[39] Silva J. E., Deus J. J. R., Calixto G. Q., Melo D. M. A., Melo M. A. F., Junior V. C. B., Chagas B. M. E., Medeiros E. P., Braga R. M. (2024). Colored
cotton crop wastes valorization through pyrolysis: a study of energetic
characterization and analytical Py-GC/MS. Sci.
Rep-Uk.

[40] Bertella S., Rana V. K., Karami P., Meinander K., Bourmaud C., Richter L., Osterberg M., Stellacci F., Pioletti D., Luterbacher J. S. (2026). Controlled
Dual Functionalization of Lignin Generates Enhanced Properties in
Gelatin-Based Hydrogels. Chem. Mater..

[41] Nicolau S. T., Matzger A. J. (2024). An evaluation of
resolution, accuracy, and precision
in FT-IR spectroscopy. Spectrochim. Acta, A.

[42] Villar L., Schlapp-Hackl I., Sánchez P. B., Hummel M. (2024). High-Quality Cellulosic
Fibers Engineered from Cotton-Elastane Textile Waste. Biomacromolecules.

[43] Mousavi S. M., Shirazi H. D., Ranta R., Asghar M. I., Kasurinen S., Halme J., Vapaavuori J. (2024). Addressing
the efficiency loss and
degradation of triple cation perovskite solar cells via integrated
light managing encapsulation. Mater. Today Energy.

[44] He J. M., Xie C. F., Long J. J. (2021). Sustainable color
stripping of cotton
substrate dyed with reactive dyes in a developed UV/K2S2O8 photocatalytic
system. J. Taiwan. Inst. Chem. E.

[45] El-Sakhawy M., Abd El-Kader A. H., Fahmy T. Y. A., Abd El-Sayed E. S., Kassem N. F. (2021). Optimization of
Dithionite Bleaching of High Yield
Bagasse Pulp. Cell. Chem. Technol..

[46] Isoaho, J. P. Dithionite Bleaching of Thermomechanical Pulp: chemistry and Optimal Conditions Doctoral Thesis; University of Jyväskylä: Jyväskylä, Finland, 2019. https://jyx.jyu.fi/bitstream/handle/123456789/64078/2/978-951-39-7768-9_vaitos_20190534_jyx.pdf

[47] French A. D. (2014). Idealized
powder diffraction patterns for cellulose polymorphs. Cellulose.

[48] Schlapp-Hackl I., Rissanen M., Ojha K., Sawada D., Gorniak K., Onkinen E., Niinimäki K., Sixta H. (2024). From postconsumer cotton
towels to new cotton textile fibers. J. Appl.
Polym. Sci..

[49] Zhao H. B., Kwak J. H., Zhang Z. C., Brown H. M., Arey B. W., Holladay J. E. (2007). Studying cellulose fiber structure by SEM, XRD, NMR
and acid hydrolysis. Carbohydr. Polym..

[50] Zhang P. P., Tong D. S., Lin C. X., Yang H. M., Zhong Z. K., Yu W. H., Wang H., Zhou C. H. (2014). Effects of acid
treatments on bamboo cellulose nanocrystals. Asia-Pac. J. Chem. Eng..

[51] Fu S., Farrell M. J., Ankeny M. A., Turner E. T., Rizk V. (2019). Hydrogen Peroxide
Bleaching of Cationized Cotton Fabric. AATCC
J. Res..

[52] Robert, R. M. ; Roger, H. W. ; Sohel, R. The Chemistry of Textile Fibres; Royal society of chemistry, 2023 10.1039/9781837670505.

[53] Balkissoon S., Andrew J., Sithole B. (2023). Dissolving
wood pulp production:
a review. Biomass. Convers. Bior..

[54] Sixta, H. Handbook Of Pulp; Wiley Online Library, 2006 10.1002/9783527619887.

[55] Asaadi S., Hummel M., Hellsten S., Härkäsalmi T., Ma Y. B., Michud A., Sixta H. (2016). Renewable High-Performance
Fibers from the Chemical Recycling of Cotton Waste Utilizing an Ionic
Liquid. ChemSuschem.

[56] Haslinger S., Wang Y. F., Rissanen M., Lossa M. B., Tanttu M., Ilen E., Määttänen M., Harlin A., Hummel M., Sixta H. (2019). Recycling of vat and
reactive dyed textile waste to new colored man-made cellulose fibers. Green Chem..

[57] Yu, C. Natural Textile Fibres: Vegetable Fibres; Woodhead Publishing, 2015; p. 29 10.1016/B978-1-84569-931-4.00002-7.

[58] Xu L. Y., Deng J. W., Guo Y., Wang W., Zhang R. Y., Yu J. Y. (2019). Fabrication of super-hydrophobic
cotton fabric by low-pressure plasma-enhanced
chemical vapor deposition. Text. Res. J..

[59] Ogrizek L., Lamovsek J., Primc G., Leskovsek M., Vesel A., Mozetic M., Gorjanc M. (2024). Improved Adhesion of
Bacterial Cellulose on Plasma-Treated Cotton Fabric for Development
of All-Cellulose Biocomposites. Molecules.

[60] Corradini E., Teixeira E. M., Paladin P. D., Agnelli J. A., Silva O. R. R. F., Mattoso L. H. C. (2009). Thermal stability
and degradation
kinetic study of white and colored cotton fibers by thermogravimetric
analysis. J. Therm. Anal. Calorim..

