# Supplementary material for: Tackling Challenges of Textile Recycling: Color Removal from Cotton Textile Waste via Sequential Reductive–Oxidative Bleaching
Source: ACS Omega. 2026 Aug 13;11(33):49496–509. doi: 10.1021/acsomega.6c02988 (PMC13520531; doi:10.1021/acsomega.6c02988)
Supplement: Supplementary file 1 [file ao6c02988_si_001.pdf]

Supporting Information

Tackling Challenges of Textile Recycling: Color Removal from Textile Waste via Sequential Reductive–Oxidative Bleaching

Shubhajit Dutta, Md. Reazuddin Repon, Inge Schlapp-Hackl, Tapani Vuorinen, and Ali Tehrani-Bagha*

Department of Bioproducts and Biosystems, School of Chemical Engineering, Aalto University, Vuorimiehentie 1, 02150, Espoo, Finland

*Corresponding author: ali.tehrani@aalto.fi

List of Content

List of Tables

Table S1. Comparative performance evaluation after color stripping of different reactive dyed cotton textile waste using different methods

Table S2. Reactive dyes with concentration (%), chemical structures, molecular formula with molecular weight, and CAS number

Table S3. Other colorimetric parameters (a*, b*, C*, h°) of undyed, dyed, and color-stripped fabrics

Table S4. Tensile strength (MPa) and elongation (%) in warp and weft orientation of the dyed and color-stripped fabrics

Table S5. Images of the dyed and color-stripped fabrics compared with the undyed fabric, generated by the X-Rite reflectance spectrophotometer

Table S6. Crystallinity index (%) of undyed, dyed, and color-stripped fabrics

Table S7. Intrinsic viscosity of undyed and color-stripped fabrics

Table S8. Temperature and mass loss pattern of undyed, dyed, and color-stripped fabrics

Table S9. Surface elemental composition (%) of dyed and color-stripped fabrics as determined by XPS

Table S10. CIE L*a*b* space and fixation efficiency of $Dyed_{Reference}$ and redyed color-stripped samples

List of Figures

Figure S1. Stepwise process diagram of reductive-oxidative color stripping.

Figure S2. Optimization of the reductive step ($Na_2S_2O_4$) by varying (a) time, (60°C, 20 g/L, pH 12, M:L=1:20), (b) M:L (60°C, 60 min, 20 g/L, pH 12), and the following oxidative step (H_2O_2) based on (c) time, (90°C, 1 g/L, pH 11, M:L=1:20), (d) M:L (90°C, 60 min, 1 g/L, pH 11).

Figure S3. Color strength (K/S) vs. wavelength (nm) plots of dyed, reductive, and reductive-oxidative stripped fabrics of (a) DG, (b) GR, (c) BL, and (d) NV.

Figure S4. CIE L*a*b* space of dyed, reductive, and reductive-oxidative stripped fabrics of (e) DG, (f) GR, (g) BL, and (h) NV compared to WH_{Ud}.

Figure S5. FT-IR primary spectra of dyed (Dy), reductive stripped (Rd) & reductive-oxidative stripped (Rd-Ox) fabric (a); spectral difference between dyed (Dy) and the other fabrics (b).

Table S1. Comparative performance evaluation after color stripping of different reactive dyed cotton textile waste using different methods

Dye(s)	Method & chemicals	Optimized condition	Color stripping%	Impact on other properties	Ref.	Remarks
Mixture of reactive dyes (see Table 1)	Sequential: 1st step: $Na_2S_2O_4$ 2nd step: H_2O_2	$Na_2S_2O_4$ 20 g/L, pH 12, M:L 1:20, 60˚C, 60 min H_2O_2 1 g/L, pH 11, M:L 1:20, 90˚C, 60 min	99.3	L* 89.1 WL% 2.2 TSL% 10.9	Current study	Mixed dyes with VS, MCT & BF based; higher CS% & lightness; comparatively lower chemical consumption; lower impact on mechanical properties
R. Red 239	Reductive: $Na_2S_2O_4$	$Na_2S_2O_4$ 40 g/L, pH 14, M:L 1:10, 80˚C, 15 min	82.9	L* N/A WL% N/A TSL% N/A	[1]	Single dye; higher chemical consumption; mechanical data not specified
Procion Turquoise HEA	Reductive: NaOH & $Na_2S_2O_4$	NaOH 10 g/L, $Na_2S_2O_4$ 10 g/L, M:L 1:15, 90˚C, 30 min	94.9	L* 73.3 WL% N/A TSL% N/A	[2]	Single dye; mechanical data not specified
R. Yellow 17	Reductive: $Na_2S_2O_4$ & NaOH	$Na_2S_2O_4$ 15 g/L, NaOH 20 g/L, M:L 1:10, 80˚C, 15 min	68.3	L* N/A WL% 3.76 TSL% 6.15	[3]	Single dye; lower CS%

R. Yellow 37	Oxidative:<br>O_3	O_3 105 mg/L, 25˚C, 45 min	89.9	L* N/A<br>WL% N/A<br>TSL% N/A	[4]	Single dye; lower CS%; mechanical data not specified
Mixture of reactive dyes	Sequential:<br>1st step: $Na_2S_2O_4$<br>2nd step: H_2O_2	$Na_2S_2O_4$ 25 g/L, pH 9, M:L 1:30, 90˚C, 90 min<br>H_2O_2 40 g/L, NaOH 20g/L, TAED 20 g/L, pH 10, M:L 1:30, 70˚C, 90 min	97.8	L* N/A<br>WL% 1.2<br>TSL% 23.9	[5]	Mixed dyes but with only VS based, Higher water, energy and chemical consumption
R. Red 228	Sequential:<br>1st step: H_2SO_4<br>2nd step: $Na_2S_2O_4$<br>3rd step: H_2O_2	H_2SO_4 10 mL/L, M:L 1:30, 60˚C, 60 min<br>$Na_2S_2O_4$ 30 g/L, NaOH 40 g/L, PVP 2.5 g/L, ND 2 mL/L, M:L 1:30, 100˚C, 60 min<br>H_2O_2 1 mol/L,	100.0	L* N/A<br>WL% N/A<br>TSL% N/A	[6]	Single dye; higher chemical consumption; longer (3 steps) process; mechanical data not specified

		NaOH 1.2 g/L, Na_2CO_3 1.8 g/L, Na_2SiO_3 7 g/L, M:L 1:30, 85˚C, 60 min				
R. Blue 19	Solvent based: NMMO & H_2O	NMMO: H_2O - 65:35, NaOH 0.5%, M:L 1:5, 95˚C, 30 min	99.0	L* N/A WL% N/A TSL% N/A	[7]	Single dye; mechanical data not specified
R. Black 5	Enzymatic: *Ganoderma lucidum IBL-05*	Enzyme 20 mL, pH 4.5, 30°C, 10 days	70.8	L* N/A WL% N/A TSL% N/A	[8]	Single dye; lower CS%; mechanical data not specified
R. Red X-3B	Photocatalytic: Nano-TiO_2/UV	TiO_2 50 mg/L, pH 8, M:L 1:30, 20˚C, 90 min	97.0	L* N/A WL% N/A TSL% 4.0	[9]	Single dye; weight loss% not specified
R. Black 5	Microwave-assisted sequential: 1st step: NaOH 2nd step: $Na_2S_2O_4$	NaOH 50 g/L, M:L 1:30, 30 s $Na_2S_2O_4$ 30 g/L, NaOH 40 g/L, M:L 1:30, 120 s	97.0	L* N/A WL% N/A TSL% 25	[10]	Single dye; higher chemical consumption; weight loss% not specified

R. Black PLS	Microwave assisted sequential:<br>1st step: NaOH<br>2nd step: $Na_2S_2O_4$<br>3rd step: H_2O_2	NaOH 8%, pH 12, M:L 1:15 2 min<br>$Na_2S_2O_4$ 30 g/L, NaOH 20 g/L, M:L 1:15, 2 min<br>H_2O_2 97.8 mL, NaOH 1.2 g/L, Na_2CO_3 1.8 g/L, Na_2SiO_3 7 g/L, M:L 1:15, 2 min	98.4	L* 88.30<br>WL% 0.1<br>TSL% N/A	[11]	Single dye; higher chemical consumption; longer (3 steps) process; strength loss% not specified
R. Red M-3BE	UV assisted reductive:<br>$Na_2S_2O_4$	$Na_2S_2O_4$ 0.6 g/L, pH 6, M:L 1:30, 20˚C, 60 min	93.3	L* N/A<br>WL% N/A<br>TSL% 11.9	[12]	Single dye; weight loss% not specified

R.: reactive, CS%: color stripping%, N/A: not available, L*: lightness, WL%: weight loss%, TSL%: tensile strength loss%, VS: vinyl sulfone, MCT: monochlorotriazine, BF: bi-functional.

Table S2. Reactive dyes with concentration (%), chemical structures, molecular formula with molecular weight, and CAS number

Reactive Dye	Chemical Structure	Molecular Formula (Molecular Weight)	CAS Number
**Reactive Red 195 (0.2%)**	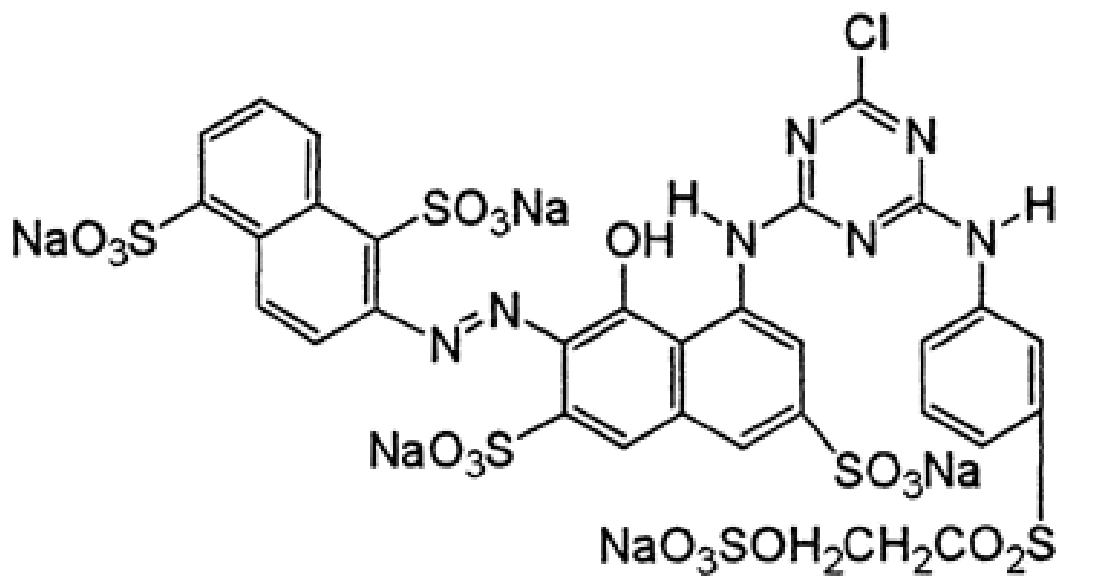	$C_{31}H_{19}ClN_7Na_5O_{19}S_6$ (1136.32)	93050-79-4
**Reactive Blue 222 (2.0%)**	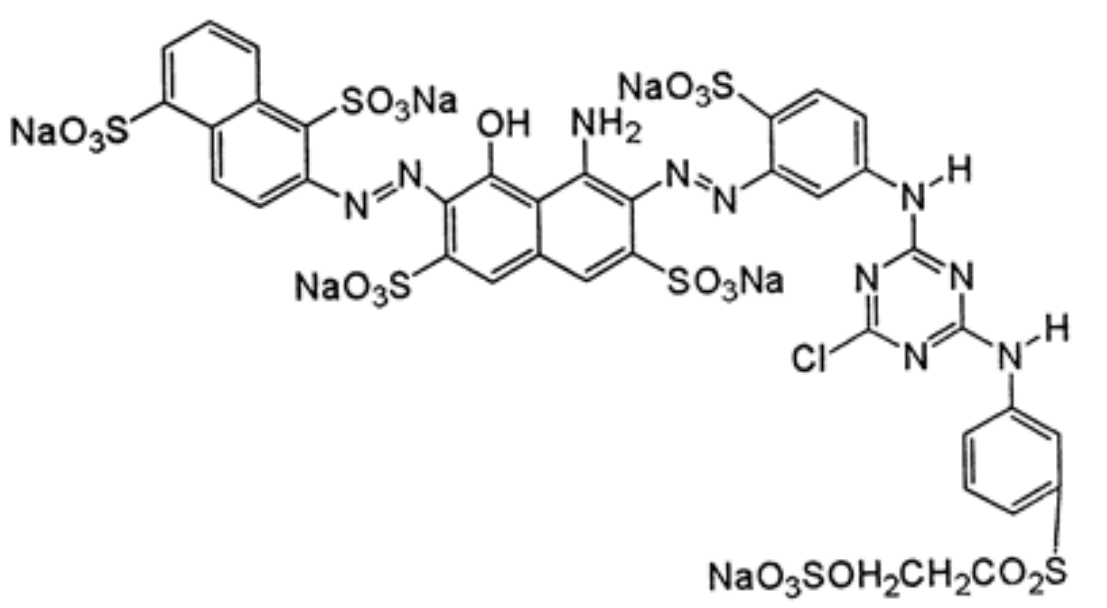	$C_{37}H_{23}ClN_{10}Na_6O_{22}S_7$ (1357.49)	93051-44-6
**Reactive Yellow 160 (1.4%)**	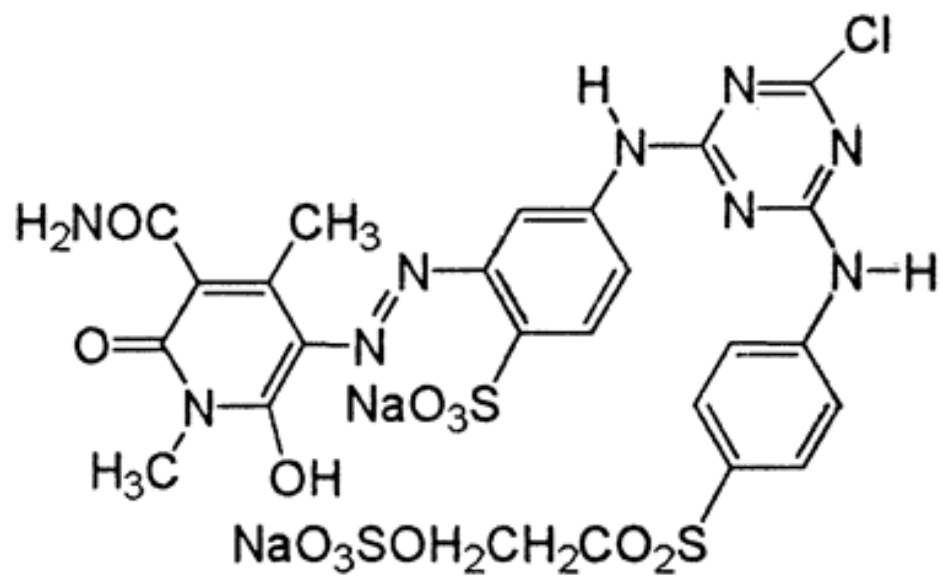	$C_{25}H_{22}ClN_9Na_2O_{12}S_3$ (818.13)	129898-77-7
**Reactive Yellow 145 (0.6%)**	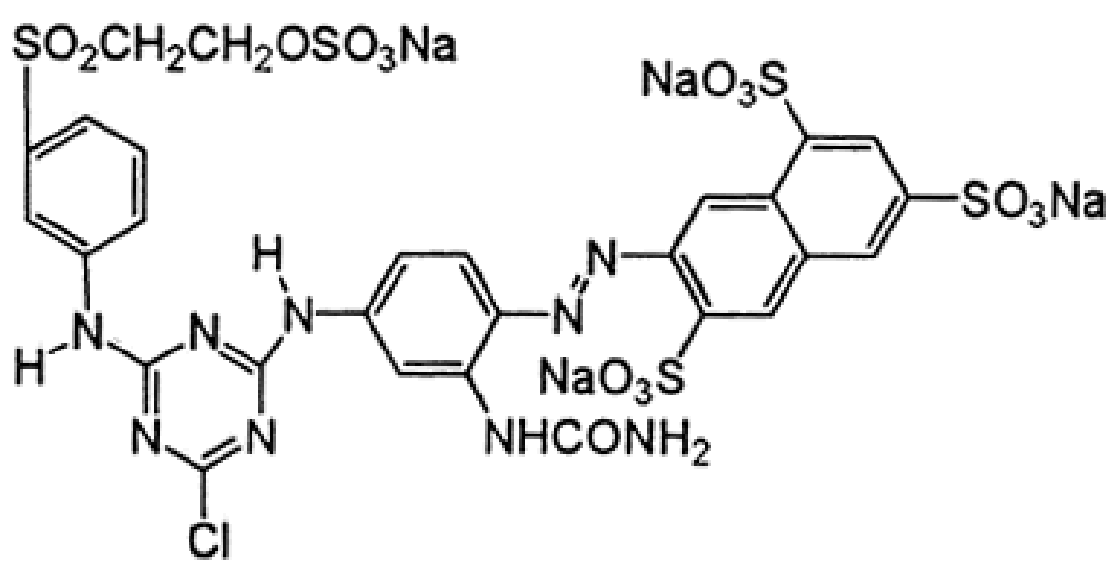	$C_{28}H_{20}ClN_9Na_4O_{16}S_5$ (1026.25)	93050-80-7

**Reactive Blue 221 (1.1%)**	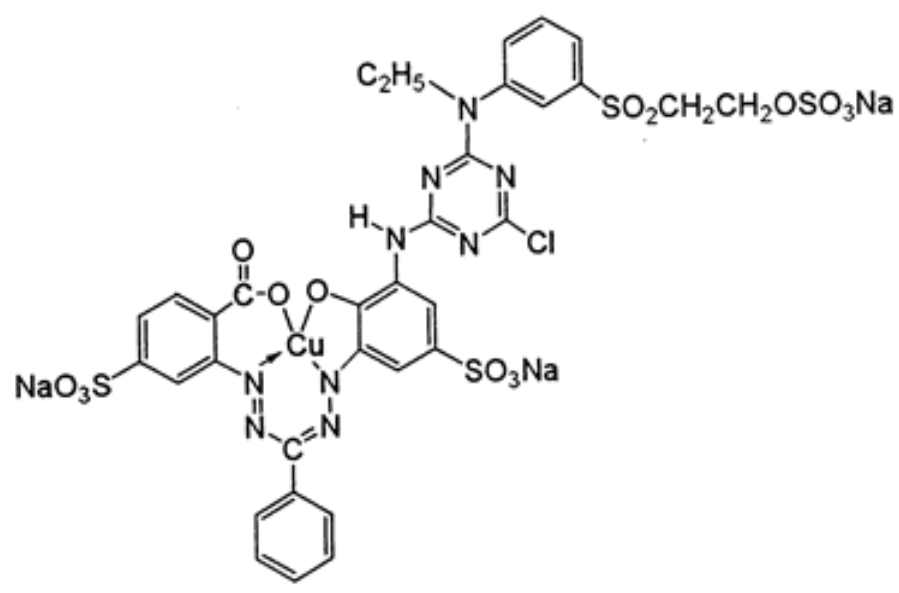	$C_{33}H_{24}ClCuN_9Na_3O_{15}S_4$ (1082.83)	93051-41-3
**Reactive Red 195 (0.6%)**	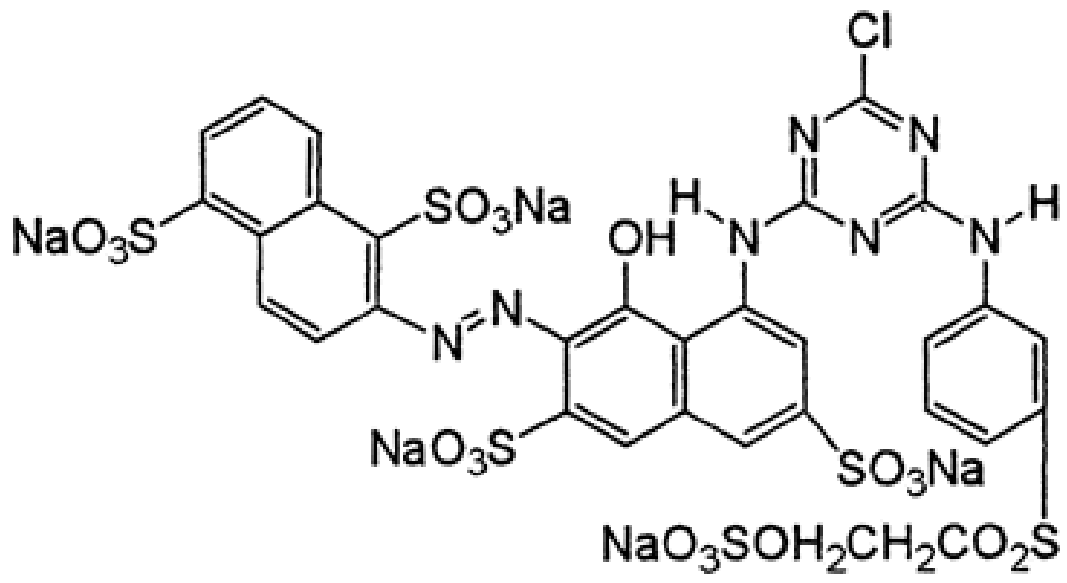	$C_{31}H_{19}ClN_7Na_5O_{19}S_6$ (1136.32)	93050-79-4
**Reactive Black 5 (2.0%)**	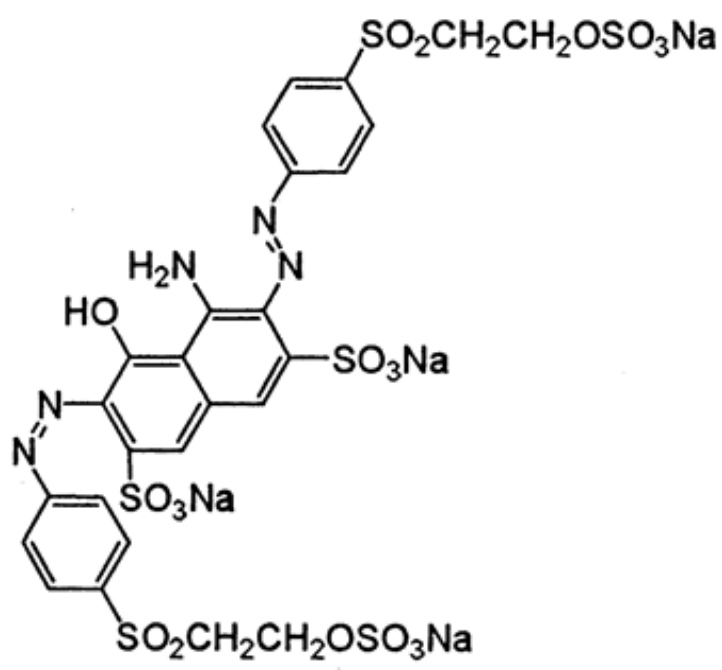	$C_{26}H_{21}N_5Na_4O_{19}S_6$ (991.82)	12225-25-1
**Reactive Orange 16 (0.3%)**	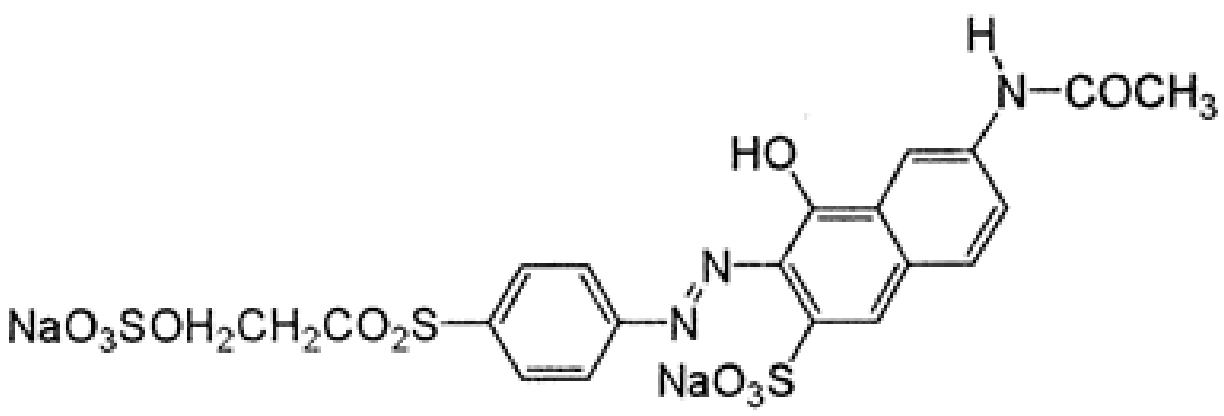	$C_{20}H_{17}N_3Na_2O_{11}S_3$ (617.54)	12225-88-6
**Reactive Orange 107 (0.2%)**	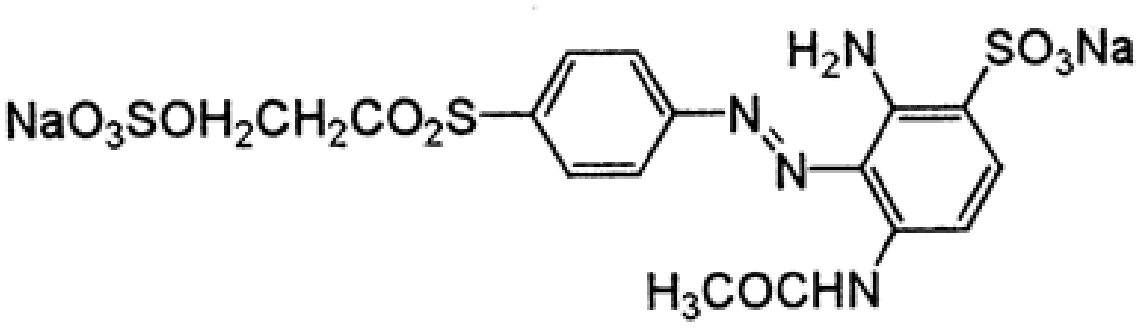	$C_{16}H_{16}N_4Na_2O_{10}S_3$ (566.49)	90597-79-8
**Reactive Black Mix (5.0%)**	Not available		

**Reactive Extra Navy D (2.2%)**	Not available		
**Reactive Red 195 (1.0%)**	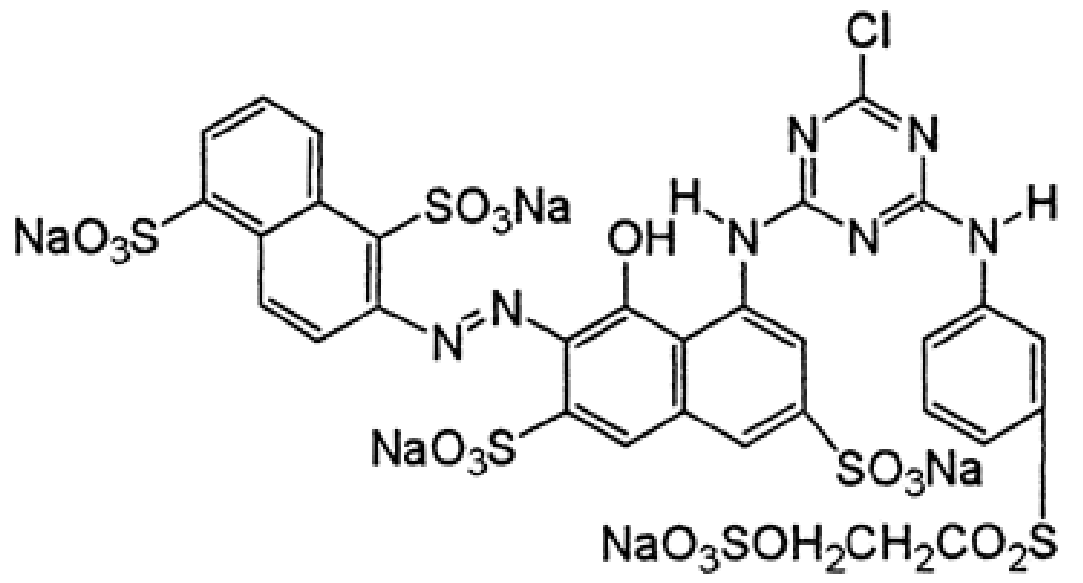	$C_{31}H_{19}ClN_7Na_5O_{19}S_6$ (1136.32)	93050-79-4
**Reactive Yellow 145 (1.3%)**	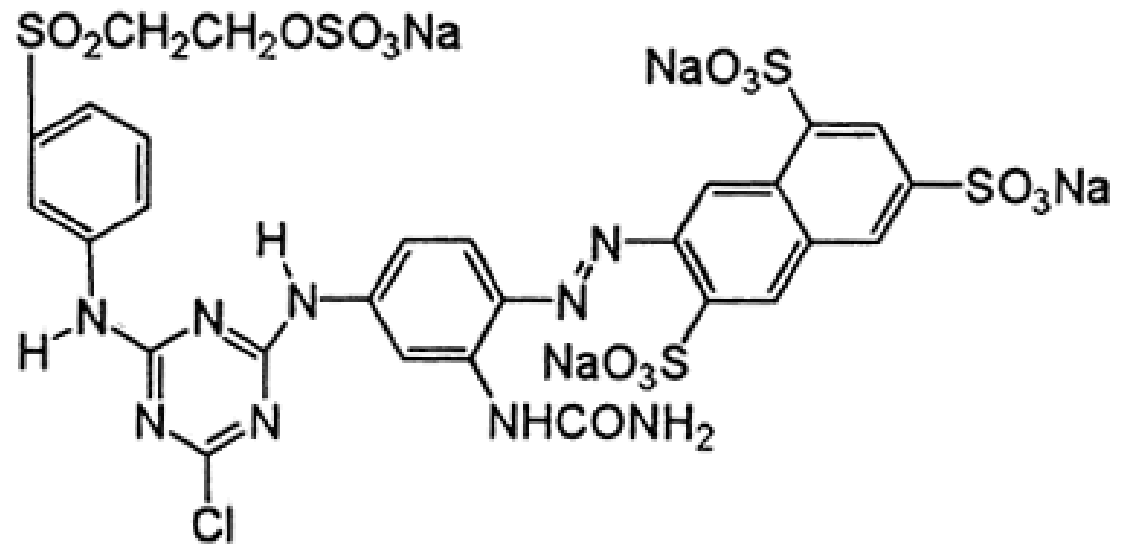	$C_{28}H_{20}ClN_9Na_4O_{16}S_5$ (1026.25)	93050-80-7

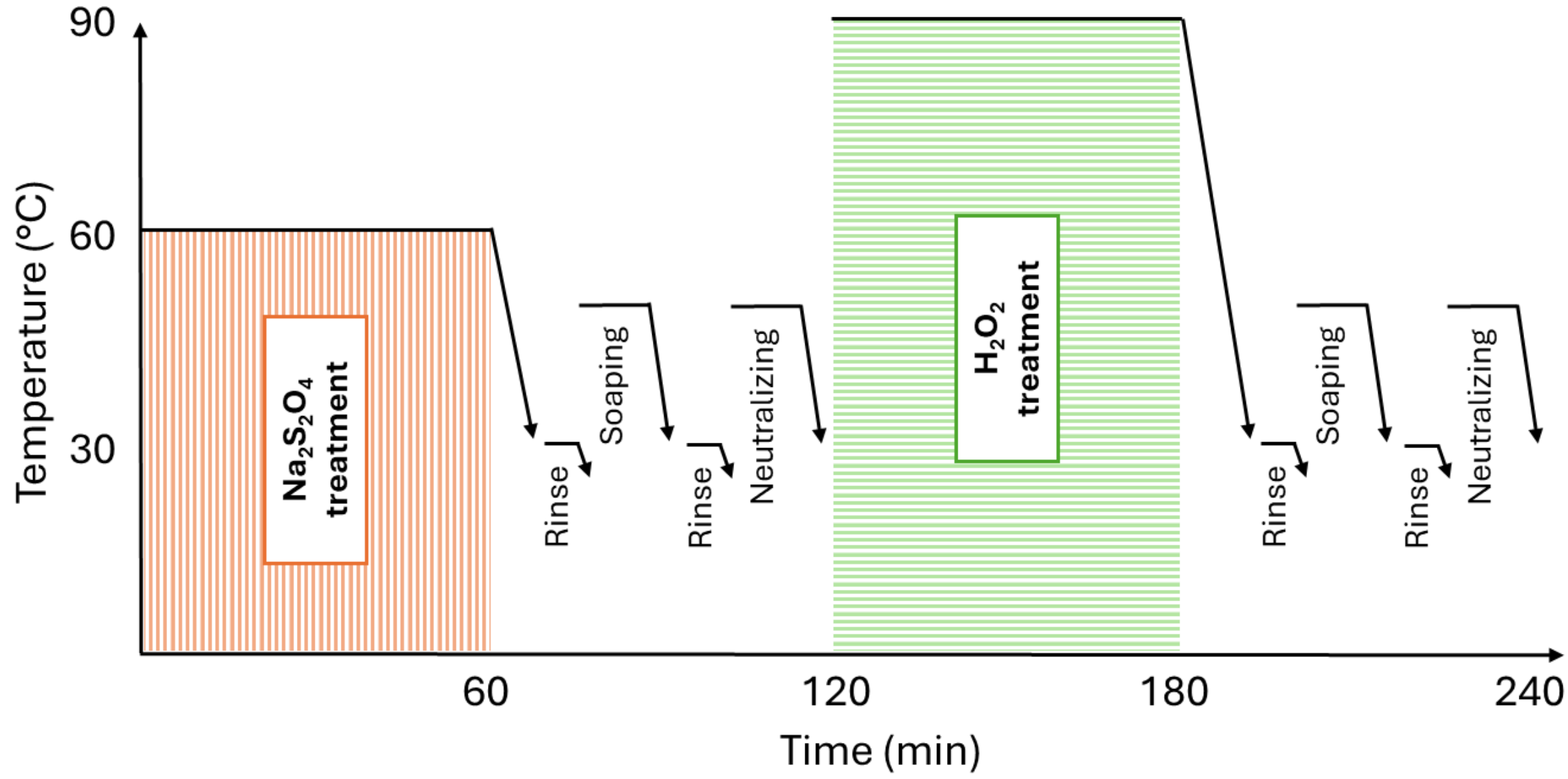

Figure S1. Stepwise process diagram of reductive-oxidative color stripping.

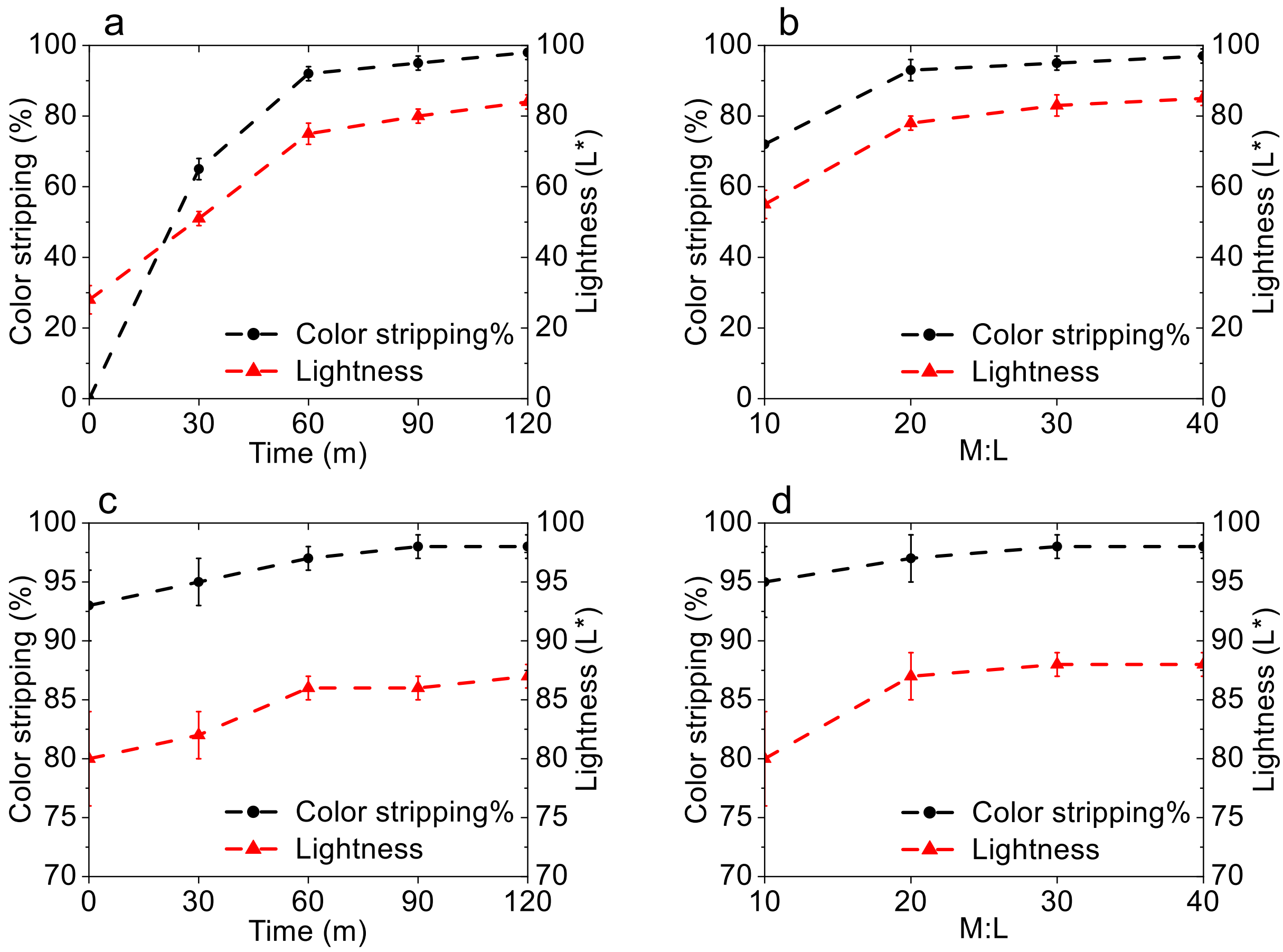

Figure S2. Optimization of the reductive step ($Na_2S_2O_4$) by varying (a) time, (60°C, 20 g/L, pH 12, M:L=1:20), (b) M:L (60°C, 60 min, 20 g/L, pH 12), and the following oxidative step (H_2O_2) based on (c) time, (90°C, 1 g/L, pH 11, M:L=1:20), (d) M:L (90°C, 60 min, 1 g/L, pH 11).

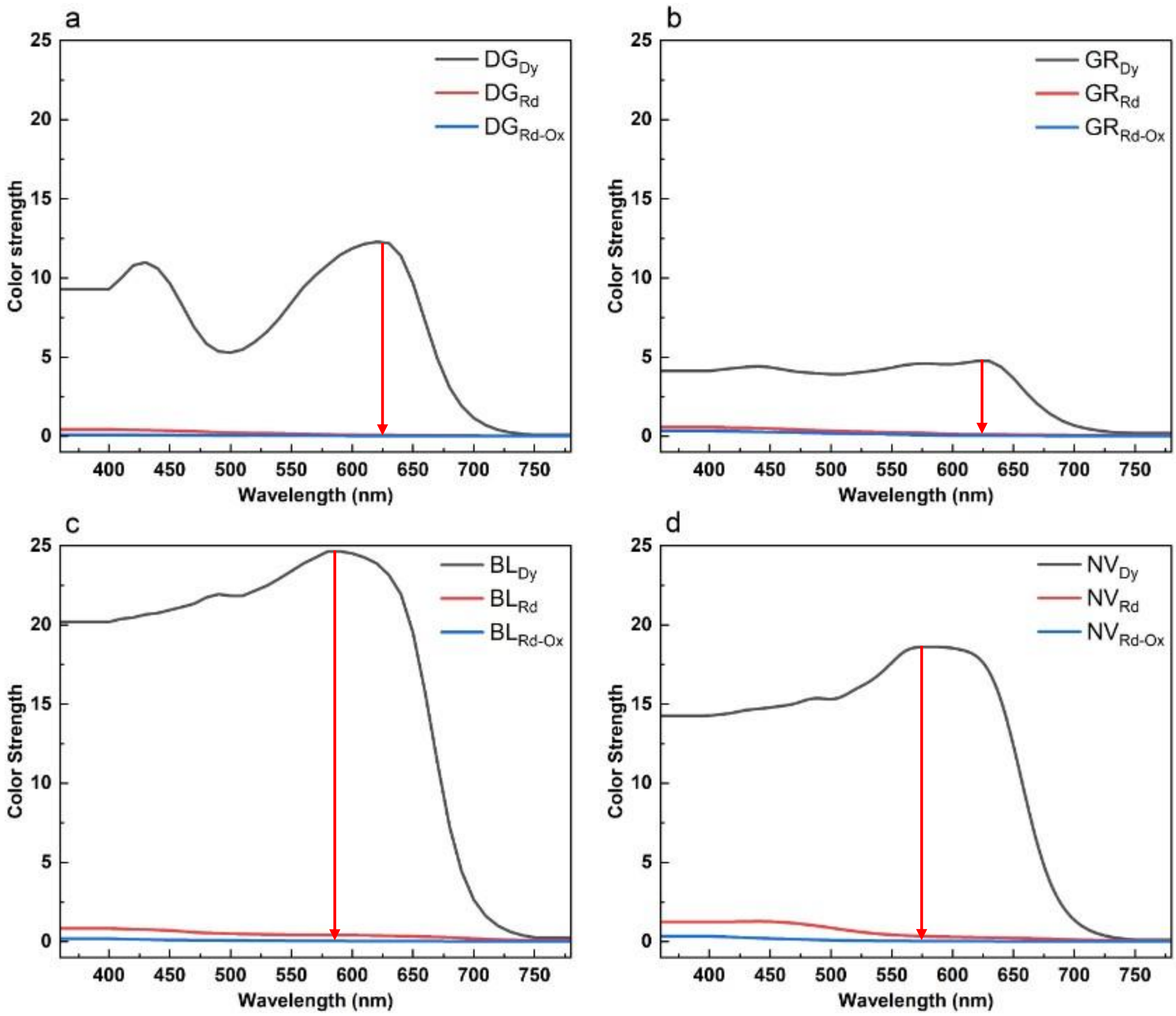

Figure S3. Color strength (K/S) vs. wavelength (nm) plots of dyed, reductive, and reductive-oxidative stripped fabrics of (a) DG, (b) GR, (c) BL, and (d) NV.

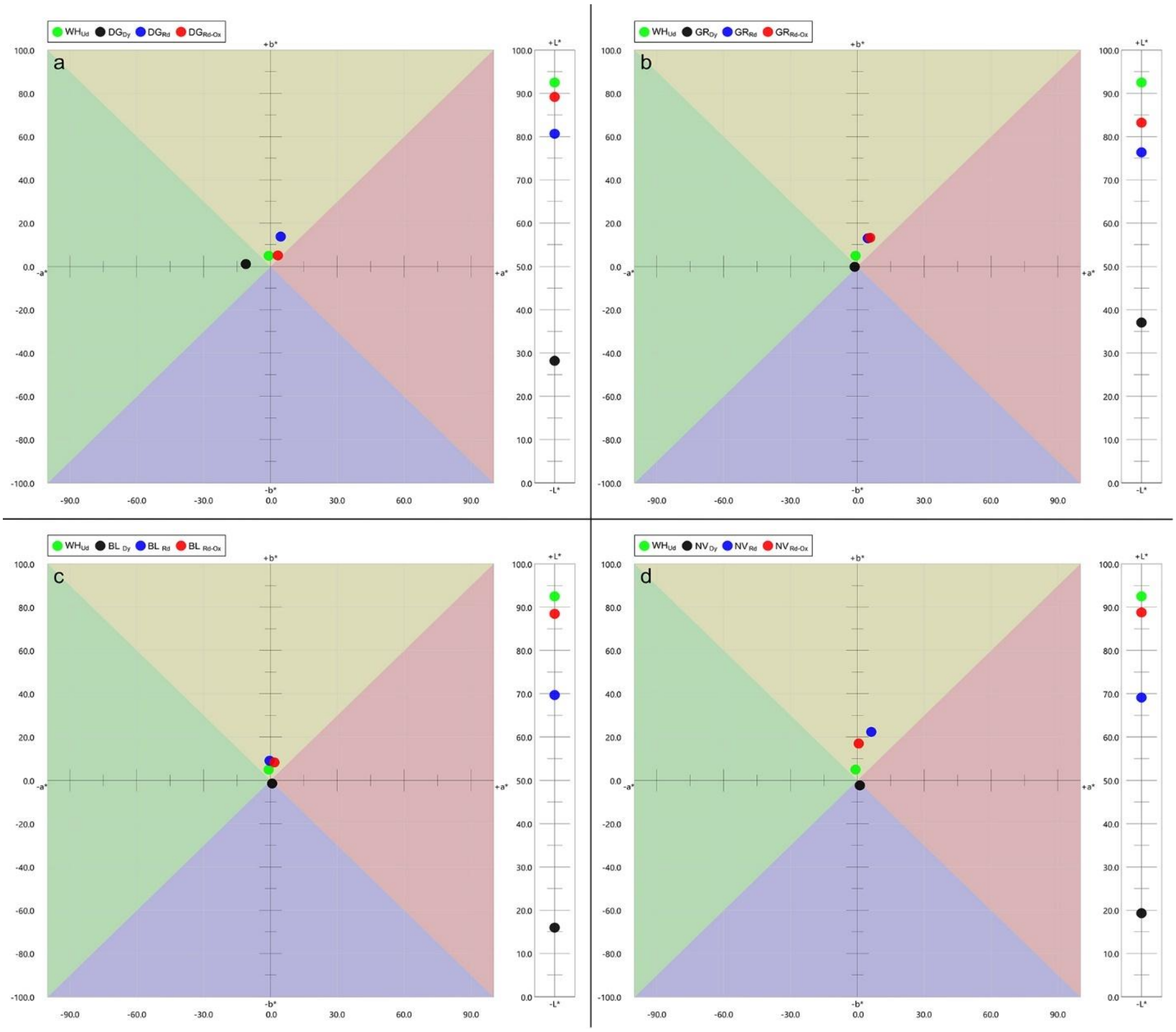

Figure S4. CIE L*a*b* space of dyed, reductive, and reductive-oxidative stripped fabrics of (a) DG, (b) GR, (c) BL, and (d) NV compared to WH$_{Ud}$.

Table S3. Other colorimetric parameters (a*, b*, C*, h°) of undyed, dyed, and color-stripped fabrics

Samples	a*	b*	C*	h°
**WH_{Ud}**	-0.81	5.04	5.11	99.07
**DG_{Dy}**	-11.1	1.19	11.1	173.9
**DG_{Rd}**	4.55	13.8	14.5	71.74
**$DG_{Rd\text{-}Ox}$**	3.29	5.17	6.13	57.54
**GR_{Dy}**	-1.24	-0.1	1.24	185.7
**GR_{Rd}**	4.67	13	13.8	70.26
**$GR_{Rd\text{-}Ox}$**	5.73	13.4	14.5	66.79
**BL_{Dy}**	0.76	-1.5	1.65	297.2
**BL_{Rd}**	-0.48	9.12	9.13	93.04
**$BL_{Rd\text{-}Ox}$**	1.79	8.31	8.5	77.85
**NV_{Dy}**	1.12	-2.3	2.58	295.6
**NV_{Rd}**	6.23	22.4	23.2	74.42
**$NV_{Rd\text{-}Ox}$**	0.59	17	17	87.99

Table S4. Tensile strength (MPa) and elongation (%) in warp and weft orientation of the dyed and color-stripped fabrics

	**Warp**		**Weft**	
**Samples**	**Tensile strength (MPa)**	**Elongation (%)**	**Tensile strength (MPa)**	**Elongation (%)**
**DG_{Dy}**	50.5 ± 0.3	16.2 ± 1.3	51.3 ± 0.3	18.6 ± 0.9
**DG_{Rd}**	45.8 ± 0.3	15.5 ± 0.7	46.5 ± 0.5	17.1 ± 0.6
**DG_{Rd-Ox}**	43.8 ± 0.9	13.0 ± 0.8	45.5 ± 0.7	15.2 ± 0.7
**GR_{Dy}**	40.4 ± 0.4	14.3 ± 0.7	39.1 ± 0.6	15.9 ± 0.6
**GR_{Rd}**	36.9 ± 0.5	13.1 ± 0.8	35.8 ± 0.4	14.3 ± 0.6
**GR_{Rd-Ox}**	36.0 ± 0.3	12.4 ± 0.6	33.6 ± 0.9	12.1 ± 0.8
**BL_{Dy}**	46.8 ± 0.8	16.9 ± 1.1	48.1 ± 0.5	18.8 ± 0.9
**BL_{Rd}**	43.2 ± 0.3	15.5 ± 0.8	43.5 ± 0.7	17.3 ± 0.3
**BL_{Rd-Ox}**	40.5 ± 0.6	13.7 ± 0.3	41.7 ± 0.9	15.9 ± 0.4
**NV_{Dy}**	58.7 ± 0.5	17.5 ± 0.7	58.1 ± 0.9	18.6 ± 0.4
**NV_{Rd}**	53.4 ± 0.3	16.6 ± 0.4	53.5 ± 0.5	17.1 ± 0.6
**NV_{Rd-Ox}**	50.9 ± 0.6	14.5 ± 0.3	50.5 ± 0.8	15.2 ± 0.5

Table S5. Images of the dyed and color-stripped fabrics compared with the undyed fabric, generated by the X-Rite reflectance spectrophotometer

	Dyed	Reductive Stripped	Reductive-Oxidative Stripped	White Undyed
**Dark Green**	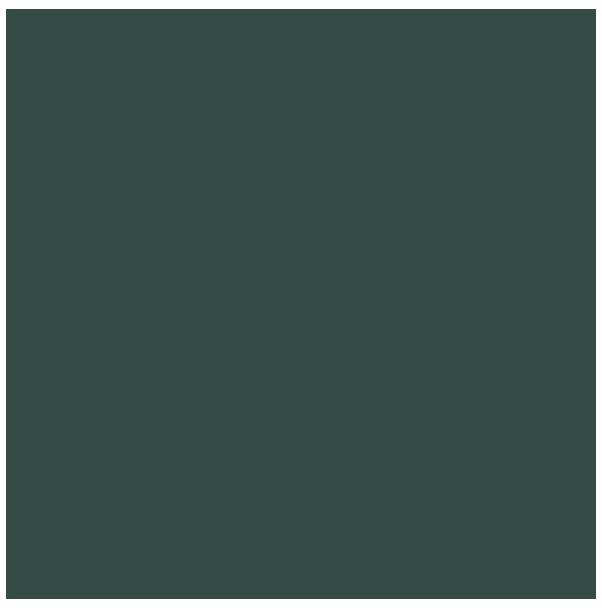	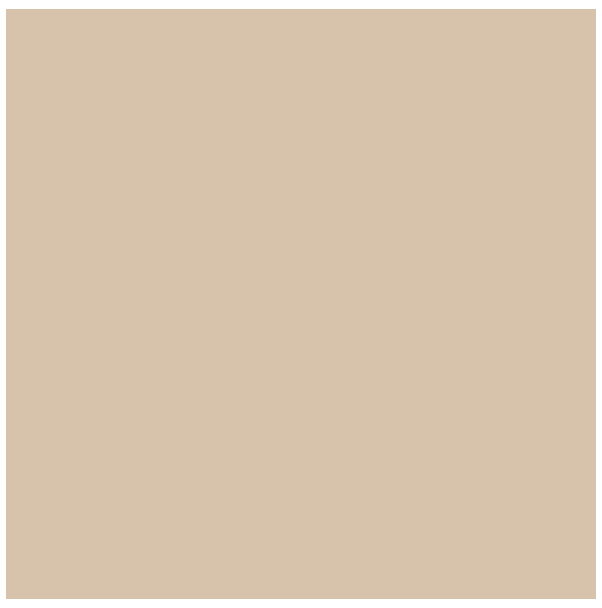		
**Grey**	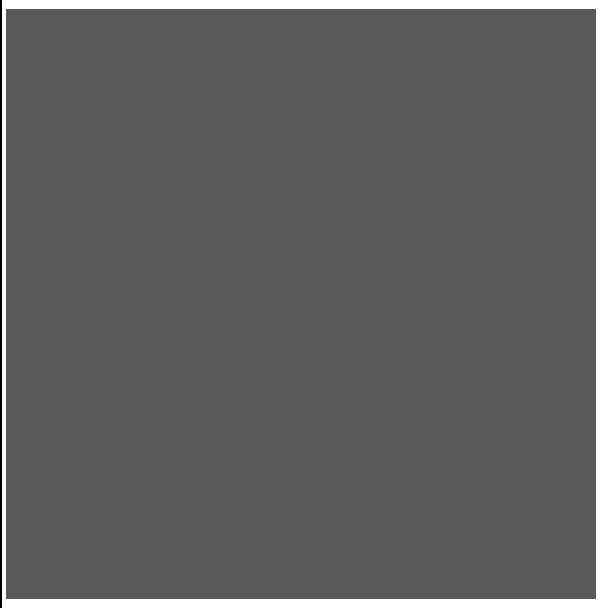	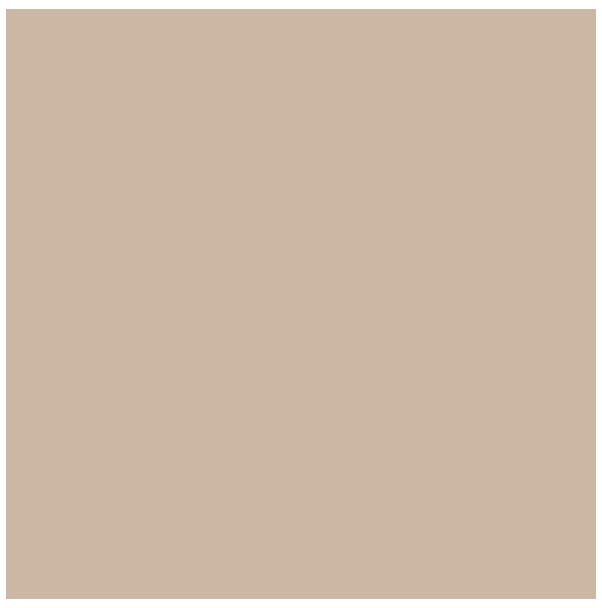		
**Black**	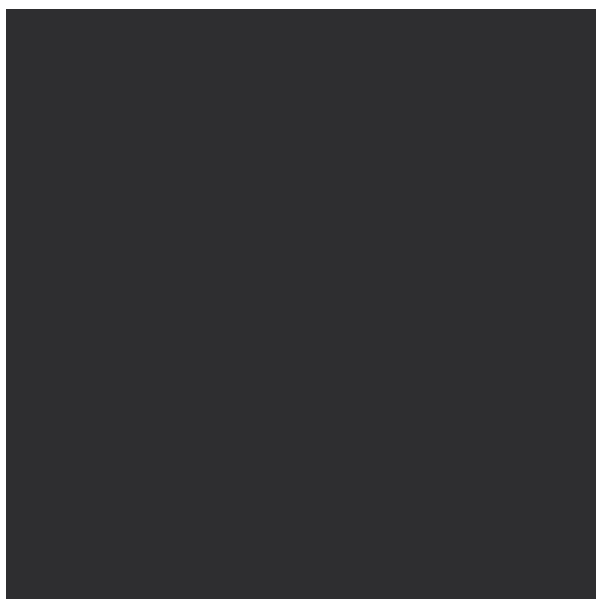	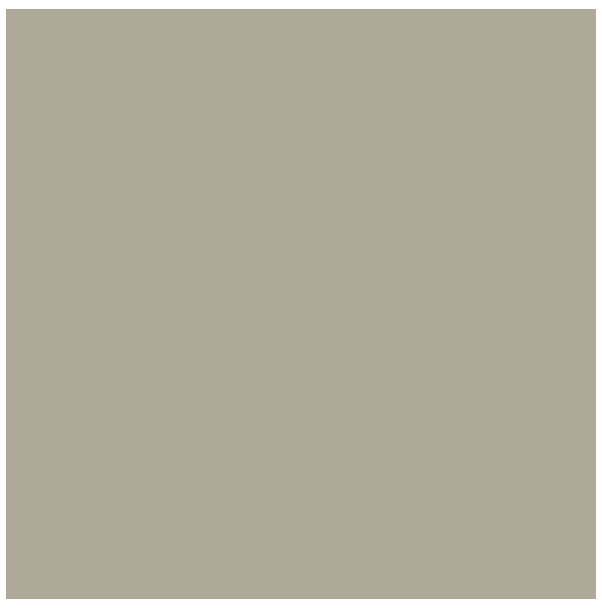		
**Navy**	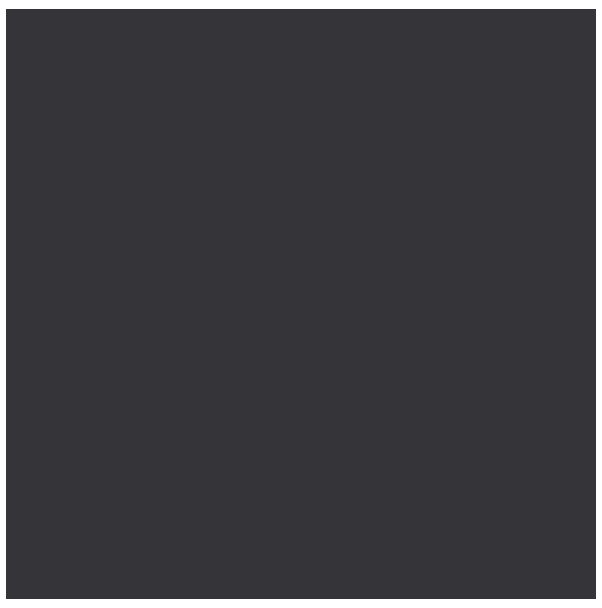	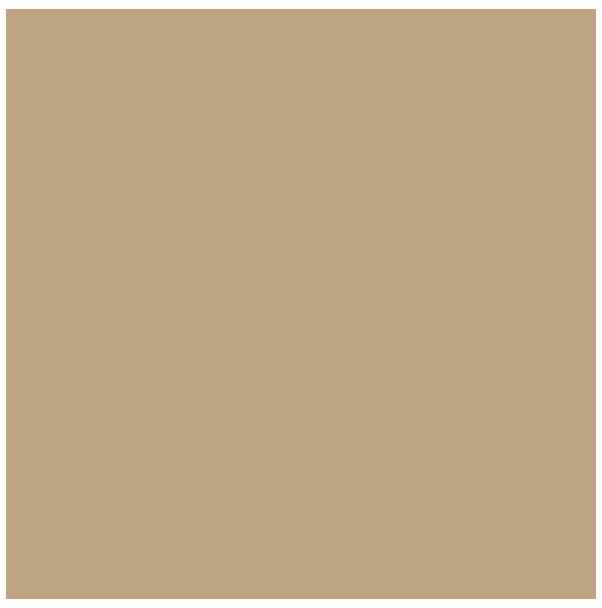		

Table S6. Crystallinity index (%) of undyed, dyed, and color-stripped fabrics

Samples	CI (%)
$\mathbf{WH_{Ud}}$	49 ± 1
$\mathbf{DG_{Dy}}$	49 ± 2
$\mathbf{DG_{Rd}}$	49 ± 1
$\mathbf{DG_{Rd\text{-}Ox}}$	53 ± 2
$\mathbf{GR_{Dy}}$	49 ± 2
$\mathbf{GR_{Rd}}$	50 ± 2
$\mathbf{GR_{Rd\text{-}Ox}}$	52 ± 2
$\mathbf{BL_{Dy}}$	47 ± 2
$\mathbf{BL_{Rd}}$	48 ± 1
$\mathbf{BL_{Rd\text{-}Ox}}$	51 ± 2
$\mathbf{NV_{Dy}}$	49 ± 2
$\mathbf{NV_{Rd}}$	50 ± 2
$\mathbf{NV_{Rd\text{-}Ox}}$	51 ± 2

Table S7. Intrinsic viscosity of undyed and color-stripped fabrics

Samples	η (mL/g)
$\mathbf{DG_{Rd}}$	775 ± 8
$\mathbf{DG_{Rd\text{-}Ox}}$	589 ± 9
$\mathbf{GR_{Rd}}$	755 ± 9
$\mathbf{GR_{Rd\text{-}Ox}}$	562 ± 7
$\mathbf{BL_{Rd}}$	804 ± 10
$\mathbf{BL_{Rd\text{-}Ox}}$	650 ± 9
$\mathbf{NV_{Rd}}$	785 ± 6
$\mathbf{NV_{Rd\text{-}Ox}}$	613 ± 8

Table S8. Temperature and mass loss pattern of undyed, dyed, and color-stripped fabrics

Samples	T_{onset} (°C)	Max mass loss (%/min)	Temperature at max mass loss (°C)	Mass loss (%)	Residual mass (%) at 600°C
WH_{Ud}	349	46	372	85	11
DG_{Dy}	306	38	330	75	16
DG_{Rd}	311	32	346	86	9
$DG_{Rd\text{-}Ox}$	333	39	357	89	7
GR_{Dy}	323	44	348	83	11
GR_{Rd}	324	37	353	87	8
$GR_{Rd\text{-}Ox}$	339	49	361	89	7
BL_{Dy}	305	37	329	71	18
BL_{Rd}	330	40	359	87	9
$BL_{Rd\text{-}Ox}$	342	47	363	87	9
NV_{Dy}	307	36	332	73	16
NV_{Rd}	322	38	352	87	8
$NV_{Rd\text{-}Ox}$	336	50	358	90	6

Table S9. Surface elemental composition (%) of dyed and color-stripped fabrics as determined by XPS

Samples	Elements			
	**C 1s (%)**	**O 1s (%)**	**S 2p (%)**	**N 1s (%)**
**Dyed fabric**	76.36 ± 0.05	21.79 ± 0.19	0.52 ± 0.03	0.51 ± 0.13
**Color-stripped fabric**	68.45 ± 0.49	31.36 ± 0.51	0.02 ± 0.01	0.12 ± 0.01

Table S10. CIE L*a*b* space and fixation efficiency (%) of $Dyed_{Reference}$ and redyed color-stripped samples

Samples	L*	a*	b*	$Fixation_{efficiency}$(%)
**$Dyed_{Reference}$**	54.4 ± 0.5	33.2 ± 0.9	12.3 ± 0.4	96.5 ± 1.2
**Redyed DG_{Rd-Ox}**	56.5 ± 0.7	30.1 ± 1.2	12.7 ± 0.8	96.1 ± 1.3
**Redyed GR_{Rd-Ox}**	58.7 ± 0.5	28.5 ± 1.0	11.9 ± 0.9	91.3 ± 1.5
**Redyed BL_{Rd-Ox}**	53.8 ± 0.4	30.6 ± 0.8	12.3 ± 0.6	96.5 ± 1.4
**Redyed NV_{Rd-Ox}**	54.8 ± 0.4	30.2 ± 1.2	14.6 ± 0.8	96.4 ± 1.3

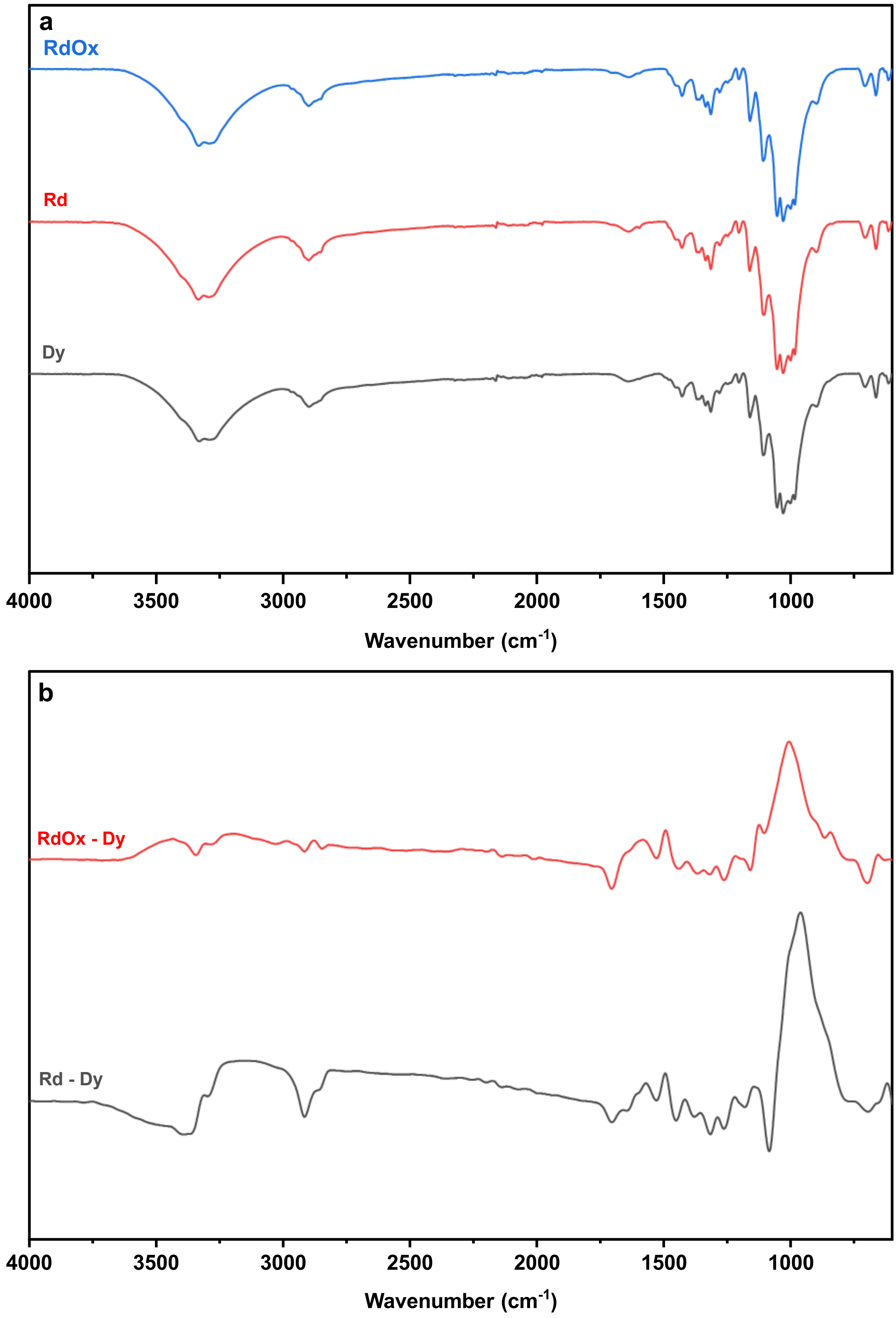

Figure S5. FT-IR primary spectra of dyed (Dy), reductive stripped (Rd) & reductive-oxidative stripped (Rd-Ox) fabric (a); spectral difference between dyed (Dy) and the other fabrics (b).

References

1. Atav, R., et al., *Determination of Color Removal and Fading Performance of Environmentally Friendly Chemical Alternatives to Hydrosulfite in Reactive Dyed Cotton Knitted Fabrics Depending on Dye Chromophore.* Journal of Natural Fibers, 2022. **19**(17): p. 15882–15890.
2. Patil, H., et al., *Solvent-based stripping method for dyed cellulosic textiles.* Journal of the Textile Institute, 2024. **115**(11): p. 2098–2105.
3. Asgher, M., Muzammil, M., Qamar, S.A., Khalid, N. and Bilal, M., *Environmentally friendly color stripping of solar golden yellow R dyed cotton fabric by ligninolytic consortia from Ganoderma lucidum IBL-05.* Case Studies in Chemical and Environmental Engineering, 2020. **2**: p. 1–4.
4. Eren, S., B. Gümüs, and H.A. Eren, *Colour stripping of reactive-dyed cotton by ozone treatment.* Coloration Technology, 2016. **132**(6): p. 466–471.
5. Li, R.J., et al., *Decolorization of dark-colored waste cotton fabric using redox decoloring agents.* Rsc Advances, 2022. **12**(28): p. 17689–17700.
6. Bigambo, P., et al., *The effect of the acid/dithionite/peroxide treatments on reactively dyed cotton and indigo dyed denim and the implications for waste cellulosic recycling.* Journal of the Textile Institute, 2020. **111**(6): p. 785–794.
7. Shao, Y.Y., et al., *Ramification of dyes for fiber-to-fiber recycling of cotton textiles.* Resources Conservation and Recycling, 2025. **222**.
8. Chatha, S.A.S., et al., *A Biological Approach for Color-Stripping of Cotton Fabric Dyed with C.I. Reactive Black 5 Using Fungal Enzymes from Solid State Fermentation.* Current Biotechnology, 2014. **3**(2): p. 166–173.
9. Long, J.J., et al., *Photocatalitic stripping of fixed Reactive Red X-3B dye from cotton with nano-TiO/UV system.* Journal of Cleaner Production, 2017. **165**: p. 788–800.
10. Younas, T., N. Tayyaba, and S. Ali, *Insights into microwave-assisted stripping of reactive black 5: a comparative perspective.* Cellulose, 2025. **32**(1): p. 629–640.
11. Zahira, T., et al., *An eco-friendly microwave assisted recycling of cellulosic fabric dyed with reactive red CL4BN and reactive black PLS.* International Journal of Biological Macromolecules, 2025. **310**.
12. He, F.N., et al., *Color stripping of reactive-dyed cotton fabric in a UV/sodium hydrosulfite system with a dipping manner at low temperature.* Cellulose, 2019. **26**(6): p. 4125–4142.
